# Exploring the miRNA Regulatory Network Using Evolutionary Correlations

**DOI:** 10.1371/journal.pcbi.1003860

**Published:** 2014-10-09

**Authors:** Benedikt Obermayer, Erel Levine

**Affiliations:** 1 Systems Biology of Gene Regulatory Elements, Max-Delbrück Center for Molecular Medicine, Berlin, Germany; Department of Physics and Center for Systems Biology, Harvard University, Cambridge, United Kingdom; University of Texas at Austin, United States of America

## Abstract

Post-transcriptional regulation by miRNAs is a widespread and highly conserved phenomenon in metazoans, with several hundreds to thousands of conserved binding sites for each miRNA, and up to two thirds of all genes under miRNA regulation. At the same time, the effect of miRNA regulation on mRNA and protein levels is usually quite modest and associated phenotypes are often weak or subtle. This has given rise to the notion that the highly interconnected miRNA regulatory network exerts its function less through any individual link and more via collective effects that lead to a functional interdependence of network links. We present a Bayesian framework to quantify conservation of miRNA target sites using vertebrate whole-genome alignments. The increased statistical power of our phylogenetic model allows detection of evolutionary correlation in the conservation patterns of site pairs. Such correlations could result from collective functions in the regulatory network. For instance, co-conservation of target site pairs supports a selective benefit of combinatorial regulation by multiple miRNAs. We find that some miRNA families are under pronounced co-targeting constraints, indicating a high connectivity in the regulatory network, while others appear to function in a more isolated way. By analyzing coordinated targeting of different curated gene sets, we observe distinct evolutionary signatures for protein complexes and signaling pathways that could reflect differences in control strategies. Our method is easily scalable to analyze upcoming larger data sets, and readily adaptable to detect high-level selective constraints between other genomic loci. We thus provide a proof-of-principle method to understand regulatory networks from an evolutionary perspective.

## Author Summary

Sequence conservation patterns can be used to assess the functional importance of a genomic locus, e.g. a binding site of a regulatory factor. If one locus is functionally coupled with another, they do not evolve independently and their conservation patterns are correlated. We used evolutionary correlations to study the vertebrate miRNA regulatory network, which is densely connected and shows rapid evolutionary rewiring. Using a Bayesian framework, we identify topological features of this network that are under weak but significant selection, such as combinatorial regulation of one gene by multiple miRNAs, or the coordinated targeting of protein complexes and signaling pathways. Our method is easily scalable to include more genomes as they are being sequenced and can be used to study correlations between other kinds of loci.

## Introduction

In the last two decades, micro-RNAs (miRNAs) have emerged as key players in post-transcriptional gene regulation [1], [2]. These noncoding RNAs have been implicated in many important pathways from development and physiology to diseases such as cancer [3]–[5]. The repertoire of miRNA genes has undergone a significant expansion in higher eukaryotes [6], in concordance with major developmental innovations along the vertebrate lineage [7]. After transcription, primary processing, and nuclear export, miRNAs are further processed by the endonuclease Dicer. The resulting 22 nt mature miRNA is loaded into the RNA induced silencing complex (RISC), which contains (among other factors) Argonaute (AGO) proteins [8].

miRNAs guide RISC to target sites in mRNA transcripts, residing mostly but not exclusively in 3′UTRs. These sites are defined predominantly via base pair complementarity to a short ∼7nt “seed” region at the miRNA 5′ end [1]. A conserved seed match is by far the most informative indicator of a regulatory interaction, but many other determinants of miRNA targeting are known, such as the sequence context in the 3′UTR, the accessibility of the site within the mRNA secondary structure, and the proximity to the stop codon or the polyadenylation site [9]. These general trends were first inferred using indirect evidence from transcriptome and proteome profiling [10], [11], and were recently corroborated by experimental advances allowing transcriptome-wide mapping of Argonaute binding sites [12]–[16], although non-canonical sites without perfect complementarity in the seed region abound [15]–[18]. Since the binding sites are short and 3′UTRs are large, typical miRNAs have potentially very large numbers of target sites across the genome. Many of these sites are evolutionarily conserved, and a major part of the transcriptome is thought to be under miRNA regulation [19].

The regulatory effect of miRNA targeting is quite diverse: the associated decrease of target mRNA levels is attributed to deadenylation followed by degradation or sequestration into P-bodies, but additional effects on protein expression result from the inhibition of translation initiation [20]. Repression of miRNA targets is usually relatively modest: typically, protein levels change by less than 2-fold [10], [11]. While the first miRNAs were identified due to their distinct function as developmental switches (*let-7* and *lin-4* in the nematode *C. elegans*) [21], [22], it has proven much harder to ascertain clear physiological or developmental roles for many of the hundreds of miRNAs discovered ever since [23]–[25]. In the known cases where miRNAs take a central and unique role in the regulatory network, the associated phenotype often seems to be conveyed by just a few out of the many predicted targets [21], [26]. In contrast to these “relevant” targets, the remaining targeting relationships appear to be either non-functional, redundant or connected to weak or subtle phenotypes. Alternatively, they could have an auxiliary role in indirectly reinforcing the functionality of the relevant target sites [27], [28]. However, distinguishing these functions by experimental or computational means has so far remained elusive [23]. In a very broad sense, miRNA regulation has been perceived mostly as an additional regulatory layer adding to the redundancy and robustness of gene expression programs [29].

With the advent of systems-level studies of gene regulation and the availability of large datasets, collective “network-level” functions of gene regulatory programs have come to be appreciated. In these cases, the function of any specific link between a regulator and its target cannot be understood without considering the regulatory context. For instance, combinatorial binding is a pervasive feature for miRNAs [30]–[32]: many genes are targeted by more than one miRNA, and often miRNAs have multiple binding sites in the same transcript. Also, since miRNAs target RNA transcripts rather than genomic DNA, the stoichiometry between regulators and targets plays an important role: the level of free miRNA is regulated by the expression of its targets, potentially leading to competitive inhibition [27], [33]–[35]. Finally, miRNAs have been implicated in coordinated regulation of entire modules of genes, such as proteins in the same complex [36] or in the same signaling pathway [37].

The best-studied examples of collective regulatory functions come from transcriptional regulation, where binding sites for entire sets of transcription factors are often clustered in *cis*-regulatory modules to integrate input from multiple regulators. While such combinatorial regulation seems to be essential for precise spatio-temporal gene expression control, it was found that transcription factor binding sites are often not strongly conserved but exhibit frequent gain and loss between species [38]–[40], with clustered binding sites evolving in a coordinated manner [41]. Since regulatory evolution was recognized as a major driving force for phenotypic change [42], [43], these particular evolutionary dynamics were intensely analyzed for signatures of adaptation [38], [44], [45]. However, alternative non-adaptive explanations are not easily ruled out [46], [47].

Here, we use covariation techniques to analyze evolutionary signatures of collective functions in the miRNA regulatory network. Generally, miRNAs have many conserved target sites, but the miRNA genes themselves, especially within the seed region, are far more conserved than these sites [2]. This has inspired the notion of an extensive rewiring of the miRNA regulatory network [2], [23], [48]. Importantly, network-level functions conveyed by more than one single target site constrain this rewiring, leading to evolutionary correlations between the gain and loss of different target sites, which means that the presence or absence of one site is correlated with the presence or absence of another site when comparing across different species. Similar techniques to utilize comparative sequence information have been employed on various genomic scales: on a small scale, compensatory mutations in homologous DNA sequences that preserve base pairing indicate evolutionary constraints due to RNA secondary structure [49]. Similarly, covariation patterns in protein sequence alignments are indicative of structural constraints [50]–[52]. On a large scale, correlations in the presence or absence of orthologous genes are attributed to common biological function [53], [54]. We hypothesized that on intermediate scales such as given by miRNA target sites, covariation patterns should offer a chance to learn features of the regulatory network from observed evolutionary correlations.

## Results

### Model

**Figure 1:**
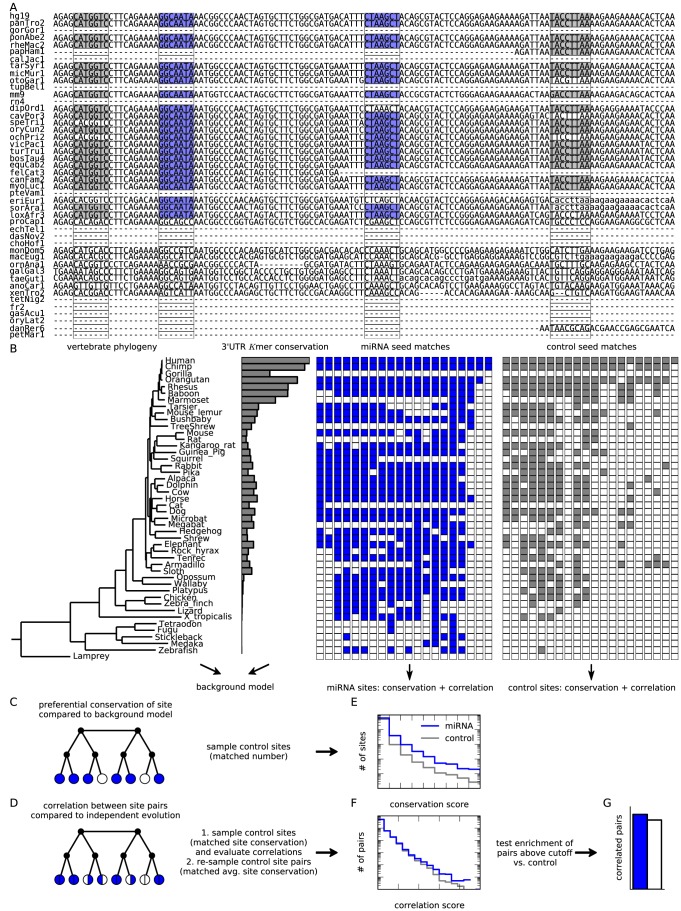
Overview of the method. **(A)** Given the 46-species vertebrate whole-genome alignment, we search for seed matches to conserved miRNA in human 3′UTRs (here the beginning of the FXR1 3′UTR is shown). **(B)** From the vertebrate phylogeny and the average 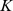
mer conservation statistics we construct a background model to serve as a gene- and species-specific prior on site conservation. Conservation patterns of miRNA seed matches (blue/white) and control seed matches (gray/white) are recorded as binary vectors (here 20 randomly distributed sites in the first 2kb of the FXR1 3′UTR are shown). These binary vectors are then used to evaluate conservation of sites and correlations between site pairs. **(C)** The background model is formulated as a Markov random field on a tree with unobserved interior nodes (black) to reproduce the average 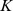
mer conservation statistics in each species and 3′UTR while accounting for the phylogeny. Site conservation is measured by comparing a model that includes a global site conservation score to the background model. **(D)** Correlations between site pairs are evaluated by comparing models with dependent or independent site pair evolution, where conservation patterns from two sites are combined into composite variables. **(E)** Conservation scores are compared to those of control seeds with a similar number of sites in human 3′UTRs. **(F)** Pair correlation scores for site pairs are found to depend weakly but significantly on the average conservation of the two sites. To avoid confounding effects from differential conservation of miRNA and control seed matches, we sample control site pairs to match miRNA sites conservation in a two-tiered strategy. **(G)** Comparing correlation scores for miRNA site pairs relative to these control site pairs, we detect enrichment of correlated site pairs for miRNA seeds.

Existing methods for miRNA target prediction using conservation signatures are based on measuring the conserved branch length along the phylogeny for each site [55]–[57], or on comparing the conservation of actual seed matches against the full empirical distribution of conservation patterns for background sites [58]. Here, our focus is not on improving target prediction but on the higher-order problem of detecting correlations in the conservation patterns of two sites. We developed a systematic, quantitative, versatile, and scalable Bayesian strategy to evaluate preferential conservation of a target site and evolutionary correlations between two target sites. Our approach, which is summarized in Fig. 1, has three essential ingredients. First, we develop a background model for the conservation of *K*mers along the vertebrate lineage, and use it to evaluate the conservation of real miRNA target sites above this background (Fig. 1**C**). Second, for each pair of sites (real or control) we calculate a pair correlation score, which measures the likelihood that the two sites evolved in a correlated rather than independent fashion (Fig. 1**D**). Finally, for different subsets of miRNAs or target genes of interest we compare the correlations among target sites with those of control sites that are matched in their conservation level (Fig. 1**E–G**). Hence, we can unambiguously attribute an excess of observed correlations to non-independent evolution specifically for miRNA seed matches.

In the analysis below we define a miRNA target site as any perfect seed match of length 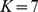 or 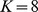 in a human 3′UTR, and record its conservation pattern in the whole-genome alignment of 46 vertebrates as a binary vector, cf. Fig. 1**A,B**. Restricting our analysis to 7mer and 8mer sites with perfect complementarity (and ignoring sites of smaller or partial seed match) lets us focus on the sites known to have relatively large conservation signal-to-noise ratios [58]. Notably, we neglect other target site features known to improve prediction algorithms [1] that would imply that site presence or absence could not be treated as a simple binary variable and require a much more complex background model. Also, we note that miRNA genes come in families defined as sets of miRNAs with the same seed sequence. Even though different family members can be expressed independently from different genomic loci and are not always functionally redundant, they have largely overlapping target sites [18]. In our simplified target site definition, we therefore do not distinguish different members of the same family. As described above, our method relies heavily on an appropriate choice of control seeds. We choose the control seeds to be as statistically similar to real seeds as possible [59]. See Methods for details.

### Background model for 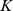
mer conservation statistics in 3′UTRs

Scoring conservation of miRNA seed matches requires an appropriate background model of *K*mer conservation in 3′UTRs across vertebrate genomes. As a starting point towards such a model we measured the average conservation of 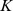
mers in human 3′UTRs in the vertebrate alignment (Fig. 1**B**). As expected, the average conservation of a 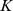
mer seen in human decreases with phylogenetic distance. However, this decrease is not only due to the sequence evolution of this site but is also influenced by other indirect features. For example, different 3′UTRs have different overall conservation levels and may even be missing or only partially alignable in some species, possibly only due to a low-coverage genome assembly. We therefore aimed to develop refined species- and gene-specific background models, by averaging the 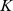
mer conservation statistics over all 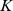
mers in a given 3′UTR and over 3′UTRs with similar conservation patterns.

Another requirement from an effective background model is to explicitly account for the phylogenetic relationships between different vertebrates. Since the existence of a site in two closely related species is more likely than in two distantly related ones, the conservation patters of two unrelated sites may seem correlated simply because they both reflect these phylogenetic (“historical”) relations. To accurately distinguish genuine evolutionary correlations from historical accidents, we designed the background model to account for phylogeny. We use a generalized phylogenetic model on the vertebrate phylogeny that not only reproduces the average frequency with which 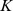
mers in a human 3′UTR are conserved in each of the other 45 species, but also how often these 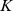
mers are simultaneously present in pairs of two other species. The pair frequencies account for the phylogenetic relationships between different species and correspond to the total branch length connecting two leaves on the tree. While as a graphical model our model is formally equivalent to a standard time-reversible phylogenetic Markov model with independent loci [60], we use a variant known as Markov random field. Its parameters correspond to branch lengths and equilibrium frequencies of a standard Markov model, with the difference that the equilibrium probability of target site occurrence is not constant along the phylogeny but decreases according to the typical pattern observed for background 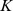
mers. This complication is required to handle correctly several types of hidden or missing data. First, alignment gaps are believed to contain evidence against site conservation, and cannot be simply discounted as missing data. Second, since we only measure the conservation of sites present in the human genome (which is used as reference for the alignment), sites that are present in other species but not in human are artificially missing from our data. Finally, the global expansion of 3′UTR length in mammals implies an apparent reduction of site conservation in other clades exceeding what is expected from neutral divergence. See Text S1 for details.

### Quantifying preferential conservation of miRNA target sites

Our background model gives the expected probability of a site's pattern of presence or absence across homologous 3′UTR positions. Functional sites are defined as those sites that are specifically conserved beyond this background. Using a maximum likelihood approach, we quantify this deviation by a conservation score 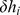 for each site 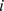. This parameter can be seen as a generalized log-odds ratio, and is conceptually related to an effective selective pressure against losing a specific miRNA target site, on top of non-miRNA-specific negative selection in this 3′UTR (see Discussion). We only consider genes with 3′UTRs alignable over a wide phylogenetic distance (from human to zebrafish), and a set of miRNA families annotated over the same distance. This restriction lets us focus on a set of presumably conserved miRNA-mRNA targeting relationships.

**Figure 2:**
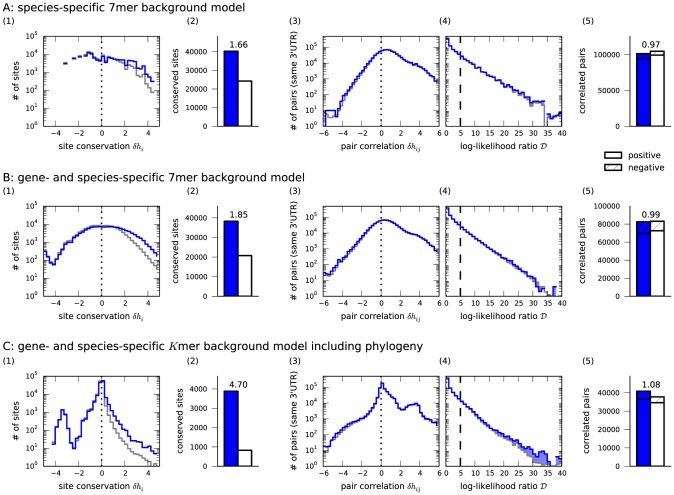
Results for different background models. To quantify conservation of miRNA target sites and correlation of site pairs we compare different background models: **(A)** species-specific (no account for phylogeny), **(B)** gene- and species-specific (no phylogeny), **(C)** full phylogenetic model with a 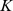
mer-specific background. Panels **(1)** show histograms of inferred values 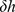 for all 7mer and 8mer seed matches (blue) vs. control seeds (gray). The peak near 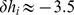 in **(C)** comes from human-specific sites. **(2)** The estimated signal-to-noise ratio at a log-likelihood cutoff of 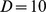 to define conserved sites increases for complex background models. **(3)** and **(4)** show histograms of inferred pair correlations 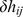 and log-likelihood ratios 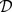 for site pairs in the same 3′UTR for miRNA target site pairs (blue) vs. matched control pairs (gray). **(5)** Only the phylogenetic background model detects a significant enrichment of evolutionary correlations among miRNA target sites at a log-likelihood cutoff of 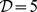 to define correlated site pairs.

Fig. 2 shows results for site conservation and correlation using background models of different complexity. A simple species-specific 7mer background model suffices to detect conserved sites above background, and a gene-specific background boosts the signal-to-noise ratio for individual sites. However, the signal-to-noise ratio for site detection reaches appreciable levels only when phylogenetic relationships between species are properly included. While we neglected other factors important for target site prediction, our method performs comparably to previous approaches in using conservation signatures [48], [58] (Fig. 2**C**). Since the signal-to-noise ratio is indeed quite modest for short (6mer) seed matches (Figure S1), we omitted these and other imperfect sites from further analysis.

Notably, the inferred values of 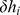 are generally not much larger than the conservation scores for the control seeds, consistent with the notion that miRNA target sites are typically not under strong selection. Of course this does not necessarily mean that these sites are not used or not functional. Low signal-to-noise ratios may be the result of weak selective pressure on the sites, unrelated selection on the 3′UTR background, or both. More interestingly, it could indicate changing evolutionary constraints due to variability in the genomic background, such as the gain or loss of other links in the regulatory network. Target sites with less isolated regulatory function or those with a supporting role would be particularly prone to reflect these events in their conservation patterns. Different from the effects of constant but weak selection, these conservation patterns would be correlated to those of other network links. To test this possibility, we analyze the correlation patterns within smaller subsets of sites with biologically plausible regulatory interactions.

### Measuring evolutionary correlations between target site pairs

By using composite variables, the phylogenetic background model can be extended in a straightforward way to model the coupled conservation statistics of two 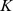
mers along the vertebrate lineage. Testing for correlations between pairs of miRNA target sites is equivalent to asking what is the likelihood that two sites did not evolve independently. To answer this question, we estimate a coupling 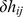 between sites 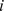 and 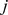 by maximizing the joint probability of observing the two conservation patterns in the coupled model. We then compare the resulting likelihood with that of an independent model where the individual likelihoods for the two conservation patterns are simply multiplied. This procedure gives the log-likelihood ratio 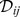 between these two models. In what follows we define two sites as correlated if 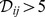, and define them as positively or negatively correlated depending on the sign of 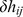. We verified that our results are not sensitive to the choice of the cutoff value (see Figure S2). We limit our analysis to sites with 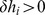; for most human 3′UTRs, this baseline corresponds to conservation across primates.

### Correlations between conservation-matched control seeds

Conservation analysis needs to carefully account for signatures of evolutionary processes unrelated to the one of interest [46]. In addition to the background model, which reproduces the average conservation statistics of 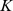
mers in each 3′UTR, we therefore used appropriately chosen control seeds (see Methods) to estimate the extent of additional variability not captured by the model. When scoring correlations between miRNA target site pairs, we found that correlation scores 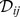 depended weakly but significantly on the average site conservation 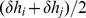 (Pearson's 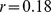), meaning that more conserved sites were more likely to appear correlated. We therefore compare the correlation between pairs of target sites with the correlations between control sites with matched conservation scores, i.e., control sites that evolve *a priori* under equally strong selective constraint. Including this control is of utmost importance when studying evolutionary correlations, since a small but non-negligible fraction of control seeds in the same 3′UTR appear correlated, especially over short distances (Figure S3). This is likely a consequence of the block structure of the multiple species alignments we used (see, e.g., Fig. 1), which is not easily incorporated into a model but implies that two 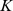
mers in the same conservation neighborhood will often have similar conservation patterns.

### The phylogenetic background model detects evolutionary correlations

Fig. 2 shows results for the correlations between sites in the same 3′UTR. Background models that do not account for phylogeny fail to detect any correlations between such site pairs above the ones seen in the control, even though a gene-specific model helps to remove spurious positive correlations arising when 3′UTRs of target genes are gained or lost entirely in certain lineages. Only the full background model is able to reliably reject false positive correlations due to shared ancestry and thus to unmask evolutionary correlations between miRNA target sites that exceed the control. Naturally, the effect is small, since conservation of the sites themselves does not strongly exceed background and contributions from pair correlations are diluted between all interaction partners. However, the highly significant excess of correlated pairs among actual miRNA seeds is thus confirmed as a miRNA-specific effect (see Methods for details on significance testing).

### Evolutionary constraints indicate conservation of combinatorial regulation

Collective functions in miRNA-mediated regulation are highlighted by the striking trend for target mRNAs to harbor more than one site for more than one miRNA [30]–[32]. Considering the often cell-type- or developmental-stage-specific expression of the miRNAs themselves [61], this strongly suggests combinatorial regulation. While the pronounced enrichment in the co-occurrence of sites for the same miRNAs compared to control sites is well-known [32], it is not clear to what extent such sites are co-conserved as an ensemble rather than independently.

**Figure 3:**
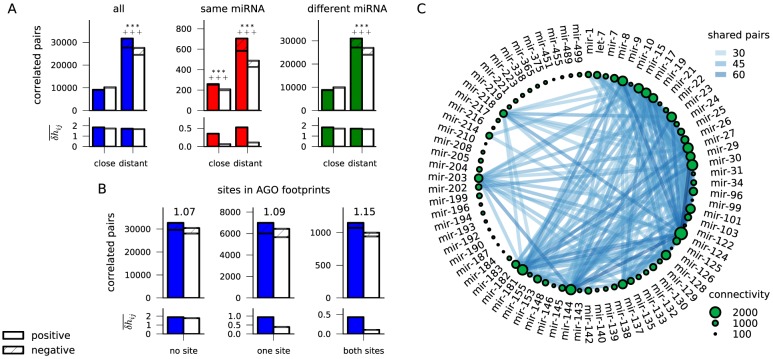
Combinatorial regulation. (A) The number of correlated site pairs (top) and mean correlation strength 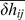 averaged over significantly correlated pairs (bottom), for close sites (distance
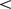
100 nt) and distant sites. Control denotes pairs of control seeds with comparable conservation, error bars (s.e.m.) from 100 bootstrap samples. Significant enrichment over control is assessed using a Poisson distribution (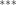: 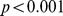). Significant excess of positive or negative (hatched) correlations is tested with a Skellam distribution (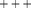: 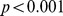 for excess positive correlations). **(B)** pair correlations with one or both sites within AGO footprints [12] have better signal-to-noise ratio (indicated on top of bars) and are more strongly correlated. **(C)** network of miRNAs with correlated target sites in the same 3′UTR; only the top 200 edges are displayed. Edge color indicates the number of correlated site pairs for two miRNAs, and the node size is proportional to connectivity (total number of correlated site pairs) for each miRNA.

We analyzed pairs of sites in the same 3′UTR and scored the number of significantly correlated pairs compared to those of control seeds (Fig. 3**A**). If two miRNAs act at the same time, cooperative effects of closely spaced sites (i.e., less than about 100 nt apart [9], [62]), which confer stronger repression than more distant sites, could also come under selection, although cooperativity is not necessary for additional selective benefits. We find that the correlation between close site pairs exceeds the background only if the sites are targeted by the same miRNA family. In this case, we also find that the average correlation strength 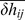 of these correlated site pairs substantially exceeds the control, indicating that regulatory links in the network are frequently strengthened via site multiplicity. The majority of the excess correlations are positive, meaning that these site pairs indeed appear simultaneously more often than expected.

While a seed match is the most informative criterion for a functional miRNA target site, various other contributing factors have been identified. Importantly, about half of Argonaute footprints detected in crosslinking assays lack a canonical seed match [12]–[16]. Also, they generally only cover a small fraction of conserved seed matches found in 3′UTRs, meaning that some conserved sites could appear so for other reasons than miRNA targeting or be used only in specific circumstances. To filter for high-confidence sites that are likely to be functional, we used data from Argonaute PAR-CLIP experiments [12]. As shown in Fig. 3**B**, site pairs that overlap with 40 nt AGO footprints (crosslink-centered regions) have higher signal-to-noise ratio and are generally more strongly correlated. We note in passing that this cross-check with orthogonal information provides further confidence that our method picks up genuine signals of evolutionary constraint.

Next, we asked whether a characterization of miRNA families could be achieved by means of interdependencies mediated through correlated target sites. We thus created a network of miRNA families by linking any two miRNAs whose target sites in the same gene are correlated. The resulting network is shown in Fig. 3**C** (Table S4). Naturally, we see a tendency for miRNA families with overlapping seeds to share more correlated site pairs (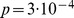 by a Mann-Whitney 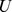 test for miRNA seeds that share 6 nt). Also, correlated sites belong preferentially to miRNAs with many conserved target sites, and these miRNAs thus have an overall higher connectivity in the miRNA-miRNA correlation network. This is especially pronounced for the miRNAs with low serial number, i.e., those that were discovered early, presumably because they are more highly and more ubiquitiously expressed and have more severe phenotypic consequences. Some interesting examples of miRNA with high connectivity include the neuronal miRNA family miR-124 [63], which is strongly connected to the similarly expressed miR-9, or the oncogenic miR-27 and miR-17. However, we also find many correlations between sites for the seemingly unrelated miR-203 and miR-144 families. On the other hand, miRNA families with relatively isolated functions include miR-126 and miR-451, which have distinct expression patterns that qualify them for use as biomarkers [61].

In line with these observations, we find that our correlation network shares significantly more edges than expected by chance with a network linking miRNAs co-expressed across different tissues [61] (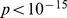 by a Fisher test; Methods), meaning that co-expressed miRNAs are more likely to have correlated target sites. Likewise, the sites of co-expressed miRNAs are enriched for pair correlations (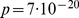, Mann-Whitney 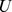 test). We also compared our correlation network to a published miRNA network linking miRNAs that target the same gene sets (such as protein complexes or signaling pathways) [37]. These two networks have more common edges than expected by chance (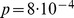), and site pairs for co-targeting miRNAs are more often correlated (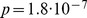). While combinatorial regulation is already evident from the co-occurrence of seed matches in the same 3′UTR, we also find that our correlation network is similar to the co-expression or co-targeting networks (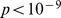 and 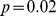, respectively) when edges are defined through the *fraction* of such co-occurring pairs that are positively correlated. Similarly, the fraction of correlated site pairs is higher for co-expressed or co-targeting miRNAs (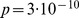 and 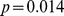, respectively). Finally, we repeated this analysis using only high-confidence miRNA target sites within AGO footprints [12]. The resulting network is highly similar to the one obtained using all correlated site pairs (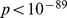 for edge overlap by a Fisher test), and accordingly we also find that co-expressed or co-targeting miRNAs are more likely to have correlated target sites (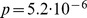 and 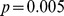 by Mann-Whitney 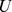 tests, respectively). Together, the strong correspondence between experimentally and computationally observed functional links between miRNAs and the selective constraints detected by our method provide an evolutionary perspective into the functionality of the miRNA regulatory network.

### Different strategies for coordinated regulation of protein complexes or pathways

Due to their large numbers of targets, miRNAs have long been considered as regulators of entire target fields, for instance by defining tissue-specific gene expression [63] or orchestrating the maternal-to-zygotic transition [64]. Associations between miRNA targets and various annotated gene sets (such as signaling pathways, protein complexes, or gene ontology categories) have been found computationally [36], [37], [58], but only very few miRNAs can be categorized uniquely in this manner [37], [58], indicating that the function of most miRNAs is less exclusive. Conversely, it has been observed that multiple components of a protein complex or a gene set are often coordinately targeted by individual or co-expressed miRNAs [36], [37].

If this coordinated regulation is indeed under selection we expect it to be reflected in evolutionary correlations. Moreover, we hypothesize that the structure of these correlations may point to an underlying regulatory strategy. For example, simultaneous targeting of the same gene by multiple miRNAs could indicate a requirement for strong repression in contrast to a fine-tuning, and would give rise to positive correlations between sites in the same gene. In contrast, a preference for simultaneous targeting of multiple genes could imply a need for a global regulatory effect and would give rise to positive correlations between sites on different genes. Finally, excess negative correlations between sites on different genes could result from a preference for a focused and local regulatory logic.

**Figure 4:**
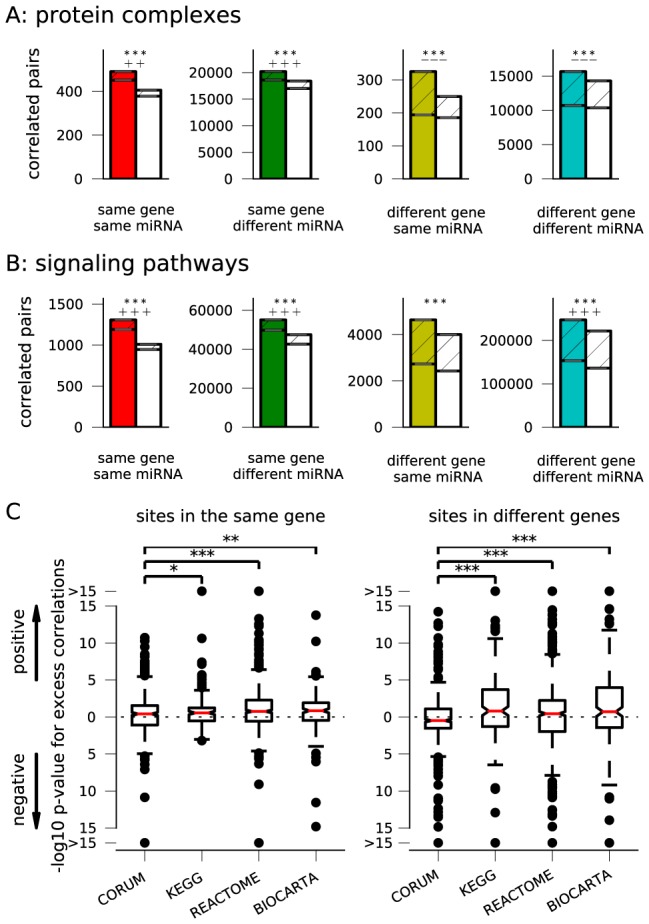
Coordinated regulation. The number of correlated site pairs in genes that encode for members of **(A)** the same protein complex (data from the CORUM database), or **(B)** the same signaling pathway (KEGG, BIOCARTA and REACTOME). Statistical significance is tested as in Fig. 3 (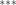: 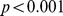, 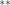: 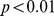, 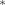: 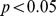; + and − for excess positive and negative correlations, respectively, with the same 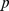-value designation). **(C)** Regulatory strategies for individual gene sets. Enrichment for positive or negative correlations between sites in the same (left panel) or in different genes (right panel) is tested and *p*-values are plotted (log-scale). Significant differences between these groups are assessed by a Mann-Whitney 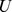 test.

We thus tested for correlations between site pairs in the 3′UTRs of genes that are members of 1878 different curated gene sets (Table S3): protein complexes from the CORUM database, and pathway sets from the KEGG, REACTOME, and BIOCARTA databases. Fig. 4**A** shows that the regulation of protein complexes is characterized by an excess of *positive* correlations between sites in the same gene (for the same or different miRNAs), but also by an excess of *negative* correlations between sites in different genes. The signaling pathways, on the other hand, show an overall excess of *positive* correlations in both cases (Fig. 4**B**). Hence, protein complexes and signaling pathways show the same pattern when it comes to correlations between site pairs targeting the same gene. However, the correlation signatures are different for site pairs targeting different genes: protein complexes tend to have excess negative correlations, possibly implying that their regulation is often implemented with a focused or local strategy. In contrast, signaling pathways have excess positive correlations, which could suggest a preference for simultaneous or global regulation of multiple members in these larger gene sets.

Since these overall trends are derived by aggregating site pairs from all gene sets, we repeated this analysis for the different gene sets individually. Sets from the four databases were tested for an excess of positive or negative correlations between sites in the same and sites of different genes (Table S5). Fig. 4**C** shows the distributions of the associated *p*-values in box plots, where data above the dotted midline indicates a preference for positive correlations and data below an excess of negative correlations. In line with our previous observations, the majority of gene sets from all categories displays an excess of positively correlated site pairs targeting the same gene, with higher significance for the signaling pathways probably because more genes are involved. However, more than half of the protein complexes show an excess of *negative* correlations between sites on different genes (the median is below the dotted line), while the majority of signaling pathways has excess *positive* correlations. Trends for individual gene sets thus confirm the global results above.

### No excess correlations between target sites for the same miRNA family

Next, we turn from a target-centric to a regulator-focused view of the miRNA regulatory network and ask if the set of target sites for the same miRNA family undergoes correlated evolution. This might be helpful to address the highly debated question to what extent miRNA-mediated regulation is influenced by competitive inhibition between different targets of the same miRNA [27], [33]–[35], [65]. Presumably, if this effect came under selection, it would lead to negative correlations between target sites on competing mRNAs. However, evolutionary signatures due to competition would be intertwined with those from global changes in miRNA functionality. For example, the loss of an entire miRNA family (or changes in its seed sequence) in a certain lineage is expected to result in global changes in selection pressure on a large set of sites of that miRNA family, lead to an accelerated turnover of these sites [58], and result in positive correlations between them. While we only considered in our analysis miRNA families conserved over large evolutionary distances, because such events clearly violate our assumption of constant selection across vertebrates, we cannot rule out more subtle changes in miRNA functionality (see also Ref. [58]).

To test for evolutionary signatures of global competition effects we analyzed correlation patterns of target sites of each miRNA family. As described above, target sites for the same miRNA in genes that encode a single functional unit show clear enrichment of correlations (Fig. 4). In contrast, a global analysis does not show such an enrichment for either positive or negative correlations, and remains inconclusive (Figure S4 **A**). A more comprehensive future study, perhaps focusing on evolutionary correlations involving specific transcripts with putative sponge functionality [34], [35], [66], [67], could help to gain a better understanding of this issue.

In order to test for signatures of changes in miRNA functionality, we stratified these results by plotting the number of positively or negatively correlated site pairs for each miRNA against the number of species where this miRNA has an annotated family member in mirBase. As shown in Figure S4 **B**, there is no detectable correlation between these two quantities. Very ubiquitiously annotated miRNAs generally have more target sites and hence possibly more correlated site pairs, but we do not see the associated positive correlation in the plot. In contrast, a negative correlation would be expected if change in miRNA functionality occurred preferentially for the miRNA families that are annotated in only few species. In addition, we also chose for each miRNA a set of control seeds with equally many and similarly conserved seed matches, tested the same number of pairs for correlation, and scored the number of correlated pairs against this control. Again, we do not detect any correlation between enrichment of positive or negative pairs and the number of annotated species (Figure S4 **C**). We conclude that given the limited statistical power of the available data, we cannot detect global signatures of correlated evolution between target sites of the same miRNA.

## Discussion

### Conceptual interpretation of conservation scores

Our Markov random field model for the background conservation statistics, that takes phylogeny into account, does not allow a direct interpretation of associated parameters (branch lengths) in terms of substitution rates. However, it offers an appealing correspondence to statistical physics, where similar models (known as Ising models) have previously been used to describe evolutionary processes [68], [69]. Notably, deep correspondences between statistical physics and evolutionary theory [70]–[72] have recently been uncovered. These approaches use Kimura's theory [73] for the fixation probability of independent rare mutations with selective advantage 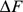 in a population of effective size 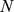. Then the expected steady-state distribution of fixed genotypes is shown to be the product of a neutral background distribution and an exponential factor for selection and drift. These two factors correspond to entropy and energy in statistical mechanics. Assuming an appropriate neutral background can be estimated, selection coefficients can be inferred by averaging over different representative samples of a population.

Our inference of a conservation score 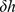 is based on the same notion of a background distribution (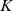
mer conservation in 3′UTRs) that is modulated by an exponential selection factor. Our estimate for 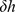 results from averaging over different species. Since genome sequences from different species do not represent independent samples, as they share a common evolutionary history, we obtain a maximum-likelihood estimate for a parameter from a set of samples by means of a phylogenetic method that accounts for this non-independence. However, the identification of our conservation score 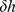 with a difference 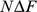 in selection between site presence and absence is strictly justified only in the limit of uncorrelated samples.

### Consistency check using orthologous sites

In the method presented here the estimate of 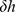 for different sites is calculated as an average over different species while accounting for their phylogenetic relationships. This average assumes that effective selection is constant along the phylogeny. Since we essentially model the outcome of complex long-term evolutionary processes including the gain and loss of entire genes, it is also required that our results should be insensitive to the specific choice of the reference species (just as unrooted trees are used for time-reversible phylogenetic Markov models). As a conceptual as well as quantitative test on these assumptions, we used the same method on a 60-way multiple species alignment to the mouse genome. We then compared inferred values for 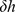 using human or mouse as reference species for more than 80000 sites at orthologous positions in the 3′UTRs of orthologous genes. The rather strong correlation in Figure S5 (Pearson's 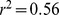) confirms that our estimates are generally robust. The slope 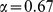 of the regression line is different from unity, probably because the baseline of background conservation (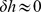) is different in mouse, where fewer very closely related genomes have been sequenced. With human as the reference species, 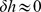 corresponds to conservation across primates. When mouse is the reference species, this baseline corresponds to conservation across rodents, i.e. over an almost 1.7-fold larger evolutionary distance than what separates primates (cf. Fig. 1**B**).

### Statistical power

The restricted available dataset of whole-genome alignments limits the obtainable statistical power. As a result, our analysis at this time could only identify global regulatory trends. However, our method is easily scalable once more vertebrate genome sequences are available, e.g., within the Genome 10K project [74]. This would significantly boost the predictive power and allow to describe the regulatory network in more detail. As a first step in this direction, we compared results obtained with a 46-species alignment to human to those obtained with a 60-species alignment to mouse (Figure S6). Naturally, the increased number of species leads to a concomitant increase in the signal-to-noise ratio, both for site conservation as well as for site pair correlations. However, this increase is yet far too modest to allow prediction of *individual* correlations. We caution that this currently suffers from a high false-positive rate, and conclusions regarding specific examples therefore warrant a more detailed analysis.

### Correlation patterns and regulatory strategies

The pattern of evolutionary correlations between sites within a functional unit may be indicative of the strategy employed in its regulation. Our data suggest that two different strategies are used in the regulation of protein complexes and signaling pathways. Excess negative correlations among target sites in different genes suggest that the control of protein complexes is focused towards a subset of the constitutive genes. In contrast, overall positive correlations among target sites in genes encoding a signaling pathway indicate that inhibition of a pathway generally requires simultaneous targeting of multiple members. Such differing trends between regulatory strategies for protein complexes and signaling pathways may reflect different functional necessities: due to their more stringent stoichiometry, downregulation of a protein complex could be achieved (perhaps even more efficiently) by strongly targeting just a few of its members: for instance, non-targeted and hence relatively more abundant members could be rapidly degraded if they are not stabilized or protected by integration into a functional complex. On the other hand, the more complicated topology and built-in redundancy of signaling pathways, which also typically contain a larger number of genes, would require inhibition at multiple control points. Otherwise, regulatory coupling between different members of a pathway could easily compensate for the downregulation of a small number of genes.

### Direct vs. indirect correlations

We point out that our method infers evolutionary correlations between pairs of target sites, which can be indicative of direct physical or functional interactions, or of indirect effects involving additional sites. Methods to disentangle the former from the latter have become very popular for aiding computational protein folding by inferring residue pairs in spatial proximity from direct contributions to the observed evolutionary correlations [51], [52]. Our approach can readily be extended towards this type of global inference, but of course this would require much larger sample sizes.

### Other regulatory factors

Our method can be straighforwardly extended to model binding sites of other regulatory factors. Interesting directions include target sites for RNA binding proteins such as Pumilio, Dnd1 or HuR which have been found to interact with miRNA targeting [75]–[77]. These sites could thus also show evolutionary correlations. Further, it has been proposed that post-transcriptional and transcriptional regulation are integrated via specific network motifs [78], [79], such that target sites of miRNAs and transcription factors would also undergo correlated evolution.

### Conclusion

The miRNA regulatory network is generally perceived as a densely connected web of relatively weak links with fast evolutionary rewiring dynamics. We reasoned that collective regulatory functions of this network constrain the rewiring patterns, and that therefore topological and functional features of the network can be inferred from the resulting evolutionary correlations. By means of a systematic, quantitative, versatile, and scalable algorithm we detect such correlations between conservation patterns of target site pairs in a specific regulatory context. Importantly, these correlations need to be distinguished from various confounding factors, among them the phylogenetic correlations between different species. Our approach achieves a reliable separation of the signal from these noise sources by means of a generalized phylogenetic model and carefully chosen controls. Our results put well-known ideas about the miRNA regulatory network, such as combinatorial regulation, on a solid evolutionary basis, and independent experimental evidence [12], [61] corroborates the functional links detected computationally. Further, we show that correlations among sites for genes in the same pathway or the same protein complex exhibit distinct trends that could reflect different control strategies. Our method serves as a proof of principle for the use of evolutionary correlations to understand regulatory networks, since it can be adapted to many different genomic loci. Notably, our generalized phylogenetic approach is an efficient coarse-grained model for the evolution of larger genomic regions, e.g., binding sites for transcription factors or RNA binding proteins, which are poorly described by explicit Markov models for individual nucleotide or amino acid substitutions. As more genome sequences become available, we expect that our approach becomes widely applicable and will be very useful to address similar questions in related fields.

## Methods

### Sequence data and annotation

Gene models of refseq genes were downloaded from the UCSC genome browser (hg19, April 9, 2013), as well as repeat masked multiple species alignments (for human: 46way alignment, Jan 17, 2012; for mouse: 60way alignment, Oct 16, 2012). For each protein-coding gene, coordinates of the longest 3′UTR isoform were extracted, and MAF blocks were extracted (“stitched”) using GALAXY tools and custom code [80], [81]. Only 7723 genes that had an annotated 3′UTR in human and zebrafish were used (see Table S1). Orthologous sites in mouse were obtained by using liftOver to map human 3′UTR coordinates to mouse.

Sequence data for miRNAs (mature.fa) and family annotations (miFam.dat) were downloaded from mirBase (Release 17) [82]. 77 conserved miRNA families with unique seeds were defined by requiring an annotated family member in human and zebrafish; seeds were extracted as the letters at position 2–8 of the mature sequences that appeared in human and the largest number of other species (see Table S2). Inconsistencies with the family set used for TargetScan [57] were resolved manually.

Members of protein complexes were obtained from the CORUM database (mips.helmholtz-muenchen.de/genre/proj/corum), and members of signaling pathways from the KEGG (www.genome.jp/kegg), REACTOME (www.reactome.org) and BIOCARTA (www.biocarta.com) databases, respectively.

### Site detection

3′UTRs of our set of genes for the reference species were searched for seed matches of 5 distinct types (oriented at the TargetScan classification [31], [57]: 8mer (or better), 7merA1, 7merm8, 6mer and offset 6mer in this order). Sites were classified as conserved in other species in the multiple alignment if the seed match was conserved identically and at orthologous positions in the alignment (see Fig. 1**A**). Site positions were recorded with respect to the 3′UTR start in the reference species. For later analysis, 7mer sites included 7merm8 and 7merA1 sites and 6mer sites included 6mer and offset 6mer sites. The data is summarized in a binary matrix 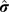 such that 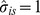 if the site 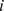 is present in a homologous 3′UTR position of species 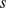, and 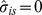 otherwise (Fig. 1**B**). We view this matrix as a sub-matrix of a larger matrix, 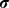, which includes not only the observed species but also their ancestors.

### Quantifying conservation of miRNA target sites

At the core of our method is the inference of site and pair conservation from sequence alignment data. The phylogenetic model gives the expected joint probability 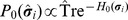 of a pattern of presence and absence across all vertebrate species (cf. Fig. 1**B,C**). Here, 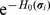 denotes the statistical weight of the given conservation pattern 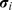 under the phylogenetic model specified through 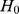. The partial trace 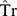 indicates that unobserved states at ancestral species are integrated out (such that 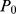 only depends on observed values 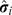; see Text S1). Functional target sites are those that are specifically conserved beyond this background. This deviation is quantified by an additional conservation score 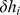 which is the same for all species but different among target sites. The optimal estimate for this parameter is found by maximizing 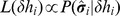, the likelihood of 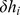 given the observed data, which in a Bayesian framework is proportional to the probability of the data given the model with parameter 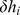. Within our formalism, this probability can be expressed as 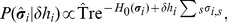
(1)where the sum in the exponent includes unobserved ancestral nodes.

### Measuring correlations

Given the conservation patterns 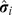 and 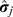 of two sites assumed to have evolved independently with respective parameters 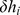 and 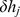, their joint probability factorizes as 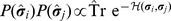 with 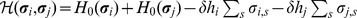 (cf. Eq. (1)). We now ask if these two patterns are better described by a joint probability that contains a coupling term 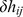: 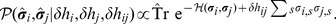
(2)The joint likelihood 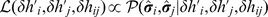 is maximized with respect to all three arguments, where the log-likelihood ratio 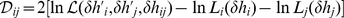 measures the significance of the observed correlation. Note that the old value 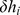 potentially contained a contribution from the coupling term that is removed in the new value 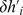 (see also Text S1). Hence, we mostly ignore the fitted values and focus preferentially on whether a site pair is correlated (choosing a cutoff of 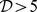; see Figure S2 for a more stringent choice), where correlations are positive if 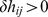 and negative otherwise. To improve the signal-to-noise ratio, we only tested pairs within much smaller subsets of sites with biologically plausible regulatory interactions (e.g., sites in mRNAs coding for members of the same protein complex). When testing all pairs of sites in a subset with more than 200 sites, we performed our analysis on 5 random subsets of 200 sites to keep the computation time manageable.

### Generation of control seeds and selection of control sites and site pairs

To generate control seeds, we first measured the dinucleotide frequencies and the histogram of the information content of the seeds of conserved miRNA families. Next we generated candidates for control seeds according to the measured dinucleotide distribution. A candidate was kept if (1) it was distinct from the set of seed sequences of any other vertebrate miRNA; (2) its reverse complement did not correspond to any of about 100 *in vitro* derived motifs for RNA binding proteins [83]; and (3) its information content 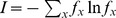 with 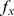 the frequency of nucleotide 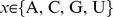 was larger than 0.4. A candidate that passed these tests was then added to the list of control seeds with probability proportional the empirical distribution of information content. We repeated this procedure to obtain a list of 5000 control seeds.

Conserved sites of real miRNAs or control seeds in Figs. 2 and S1 are defined as sites with a log-likelihood ratio of 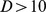. Estimating the signal-to-noise ratio is done by dividing the number of conserved sites of a real miRNA by the average number of conserved sites of a corresponding subset of control seeds. This corresponding subset is obtained by selecting from the list of 5000 control seeds only those that have a similar number of seed matches as the real miRNA (±15%) in human 3′UTRs [32]. This was done separately for each site type (6mer, 7mer, 8mer).

As control for the pair correlations observed in the genes of a functional unit, we select conservation-matched control sites in those genes. For each miRNA that has a target site in this functional unit we choose a set of control seeds with a similar number of sites, and a similar distribution of conservation scores. More specifically, for each targeting miRNA we generate the histogram of the 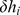-values of its 7mer and 8mer target sites using 10 equipopulated bins. We then select the 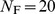 control seeds that have the most similar histogram for target sites in these genes (using the relative squared difference of the bin counts), after removing control sites that overlap any real site. The ordering of these control seeds is randomized to avoid creating a hierarchy in the sets of control sites. All sites associated with the selected control seeds are then used for pair correlation analysis.

In a second step, we use a biased bootstrap approach to make the ensemble of control site pairs even more similar to the real site pairs. For the sites in each category (e.g., all site pairs, or site pairs for the same miRNA), we collect the mean values 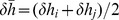 of the sites in each pair, lumping together pairs from all the 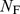 sets of control sites. We then create a histogram of 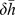-values for pairs in each category using 10 equipopulated bins. Pairs of control sites are re-sampled into 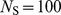 bootstrap samples with a probability that is proportional to the ratio of bin counts of the real vs. control site pairs. Because this does not always give strictly equal numbers of real and control site pairs, we re-scale the number of significant control site pairs according to the size of the bootstrap samples. Figure S7 shows that this method gives very similar distributions of average site pair conservation, while the pairs with 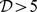 are shifted towards stronger conservation. Here we also compare to an unbiased bootstrap using a uniform probability in the re-sampling step.

In the cases where there are more than 200 sites in the set under consideration, we randomly choose 5 sets of 200 sites each, because calculating 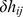 for all ∼20000 pairs is computationally very expensive. The analysis is done for each set independently, and results are averaged at the end. All *p*-values are reported as the median over the 5 sets.

### Significance estimation

We observed that the statistics of significantly correlated site pairs (with 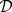 exceeding the cutoff) for the control seeds is compatible with a Poisson distribution, because the variance over the bootstrap samples is strongly correlated and scales linearly with the mean (see Figure S8 **A**). Hence, we used the Poisson distribution to test the significance of an enrichment of correlated pairs relative to the mean of the bootstrap samples in the control. For detecting an excess of positive or negative correlations, we found that the numbers of significantly correlated pairs with positive or negative correlations, respectively, were entirely uncorrelated when comparing across bootstrap samples (Figure S8 **B**). Therefore, we treated these values as independent Poisson variables, and used the Skellam distribution for their difference to test for an excess of positive or negative correlations compared to control.

For comparing the miRNA-miRNA correlation network of Fig. 3**C** to the co-expression or co-targeting network, we extracted expression information for members of the miRNA families used here from 172 different RNA libraries from major organs and cell types summarized on microRNA.org (based on the expression atlas of Ref. [61]). Read counts for all family members were summed up, and overlapping expression between miRNA families was quantified by a normalized dot product of the expression values across the different tissues. For our correlation network, we define an edge between miRNA families if it is in the top 50% of edges. Similarly, we calculate the fraction of correlated edges by dividing by the number of site pairs tested, and define edges in this network from the top 50% of connections. For the co-expression network, we use a corresponding cutoff on the expression overlap between two miRNAs. Other percentile cutoffs to define edges give largely similar results. The co-targeting network was extracted from Ref. [78] using their significance cutoffs to define edges. We then test network similarity by means of Fisher's exact test for the number of shared vs. distinct edges, and enrichment for correlations for target sites of co-expressed or co-targeting miRNAs by means of a Mann-Whitney 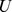 test.

## Supporting Information

Figure S1
**Site conservation statistics.** **(A)** Histogram of inferred values 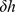 for 6mer, 7mer, and 8mer seed matches with 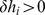 (solid) vs. control seeds (dashed). **(B)** Estimated signal-to-noise ratio (compare shaded area in **A**) at a log-likelihood ratio 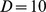.(PDF)

Figure S2
**Cutoffs on** 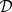 **.** Results as in Fig. 3**A** (panel **(A)**), Fig. 4**A** (panel **(B)**) and Fig. 4**B** (panel **(C)**), but for a cutoff 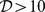 to detect significantly correlated pairs. While the number of correlated pairs decreases, and negative correlations are more frequent, none of our conclusions is changed, demonstrating the robustness of our results to the arbitrary choice of the cutoff value.(PDF)

Figure S3
**Correlations between control seeds.** Even with the full phylogenetic 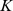
mer background model, a small fraction of control seed pairs in the same 3′UTR shows distance-dependent, mostly positive, correlations.(PDF)

Figure S4 **Pairs for the same miRNA.** **(A)** shows that correlations between site pairs for the same miRNA (but mostly in different 3′UTRs) are not found to exceed the control. However, this analysis can be used to test for signatures of changes in miRNA functionality. **(B)** number of positively (+) and negatively (×) correlated pairs for each miRNA as a function of the number of species where this miRNA is annotated. There is no significant Spearman correlation as indicated below the plot. **(C)** Scoring enrichment in the number of positively or negatively correlated pairs relative to matched control seeds gives similar results.
(PDF)

Figure S5
**Conservation of orthologous sites.** Comparison between inferred values for roughly 80000 orthologous sites using human or mouse as reference species shown as density plot. Solid line indicates regression (Pearson 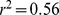, slope 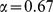), dashed line diagonal.(PDF)

Figure S6
**Analysis of statistical power.** To assess statistical power of our method, we compare results for a 46-species alignment to human to results using a 60-species alignment to mouse. **(A)** shows that significantly conserved 7mer and 8mer sites (at a log-likelihood-ratio of 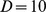, compare Fig. 2**C** **(2)**) for miRNA seed matches (blue) and control seeds (white). Signal-to-noise ratio is indicated on top of the bars and increases by 14% when the number of species increases by 30%. **(B)** Significantly correlated site pairs at a log-likelihood cutoff of 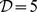 as in Fig. 2**C** **(5)**. Signal-to-noise ratio increases by 6% when increasing the number of species.(PDF)

Figure S7
**Control seeds.** Generating pairs of control seed matches entails selecting control seeds with similar conservation as miRNA seeds (here for the data shown in Fig. 2**C**). **(A)** We compare the histograms for the average conservation 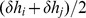 of each pair of control seeds (dotted) against pairs of actual miRNA seeds (solid), and use a biased bootstrap to enrich for pairs with similar conservation (dashed, on top of solid). Correlated pairs (red) are on average more conserved than this ensemble. We also checked that the histograms for the difference in conservation 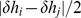 (**B**), and for the site distance (**C**) are matched.(PDF)

Figure S8 **Statistics of correlated site pair occurrence.** **(A)** the number of correlated site pairs for control seeds in the same 3′UTR behaves like a Poisson variable where the mean equals the variance (each dot is a 3′UTR; linear regression on log values). **(B)** the mean numbers of positively or negatively correlated site pairs per 3′UTR are not correlated (each dot is one bootstrap sample).
(PDF)

Table S1 **List of genes used in this study.**
(XLSX)

Table S2 **List of miRNA families used in this study.**
(XLSX)

Table S3 **List of 1878 curated gene sets and their members.**
(XLSX)

Table S4 **miRNA-miRNA correlation network.**
(XLSX)

Table S5 **List of gene sets with significant enrichment of correlated site pairs at 5% FDR.**
(XLSX)

Text S1 **Supplementary methods.**
(PDF)
