# Correction: Exploring the miRNA Regulatory Network Using Evolutionary Correlations

**DOI:** 10.1371/journal.pcbi.1004015

**Published:** 2014-11-11

**Authors:** The PLOS Computational Biology Staff

The affiliations of this manuscript were incorrectly listed. The correct affiliations are:

Benedikt Obermayer^1,2^, Erel Levine^2^

^1^ Systems Biology of Gene Regulatory Elements, Max-Delbrück Center for Molecular Medicine, Berlin, Germany

^2^ Department of Physics and Center for Systems Biology, Harvard University, Cambridge, USA

The funding statement is incorrect and should read as follows:

This work was supported by fellowships of the German Academic Exchange Service (www.daad.de) and the MDC (www.mdc-berlin.de) (to BO), and the National Science Foundation (www.nsf.gov) through grant MCB-1121057. The funders had no role in study design, data collection and analysis, decision to publish, or preparation of the manuscript.
