# Supplementary figures and images for: Exploring the miRNA Regulatory Network Using Evolutionary Correlations
Source: PLoS Comput Biol. 2014 Oct 9;10(10):e1003860. doi: 10.1371/journal.pcbi.1003860 (PMC4191876; doi:10.1371/journal.pcbi.1003860)

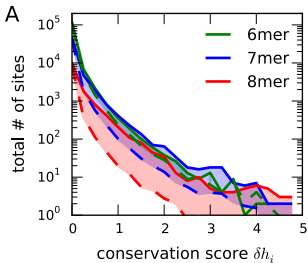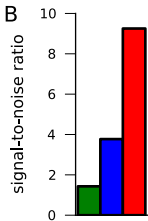

Supplement: Figure S1 — Site conservation statistics. (A) Histogram of inferred values for 6mer, 7mer, and 8mer seed matches with (solid) vs. control seeds (dashed). (B) Estimated signal-to-noise ratio (compare shaded area in A) at a log-likelihood ratio . (PDF) [file pcbi.1003860.s001.pdf]

# A: sites in the same 3'UTR

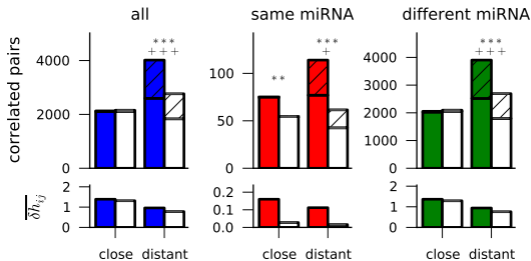

# B: protein complexes

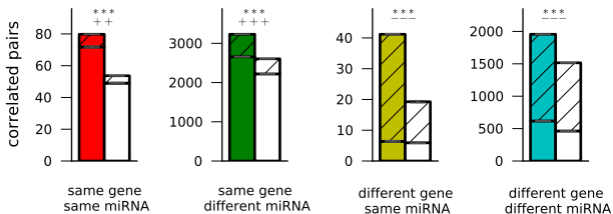

# C: signaling pathways

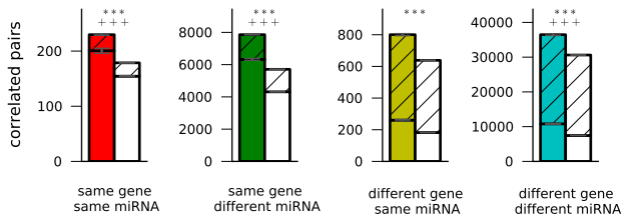

Supplement: Figure S2 — Cutoffs on . Results as in Fig. 3A (panel (A)), Fig. 4A (panel (B)) and Fig. 4B (panel (C)), but for a cutoff to detect significantly correlated pairs. While the number of correlated pairs decreases, and negative correlations are more frequent, none of our conclusions is changed, demonstrating the robustness of our results to the arbitrary choice of the cutoff value. (PDF) [file pcbi.1003860.s002.pdf]

# background correlations

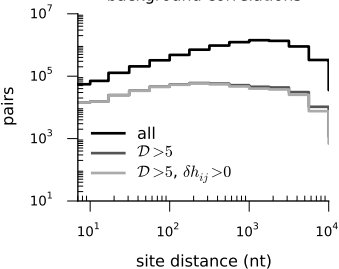

Supplement: Figure S3 — Correlations between control seeds. Even with the full phylogenetic mer background model, a small fraction of control seed pairs in the same 3′UTR shows distance-dependent, mostly positive, correlations. (PDF) [file pcbi.1003860.s003.pdf]

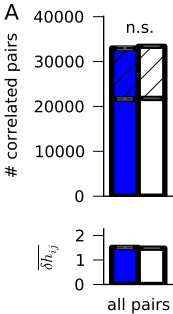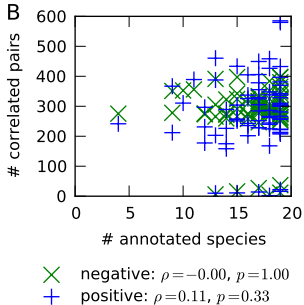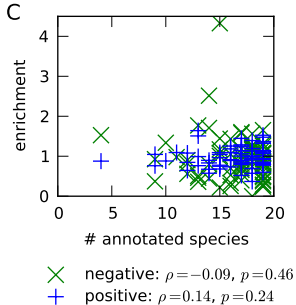

Supplement: Figure S4 — Pairs for the same miRNA. (A) shows that correlations between site pairs for the same miRNA (but mostly in different 3′UTRs) are not found to exceed the control. However, this analysis can be used to test for signatures of changes in miRNA functionality. (B) number of positively (+) and negatively (×) correlated pairs for each miRNA as a function of the number of species where this miRNA is annotated. There is no significant Spearman correlation as indicated below the plot. (C) Scoring enrichment in the number of positively or negatively correlated pairs relative to matched control seeds gives similar results. (PDF) [file pcbi.1003860.s004.pdf]

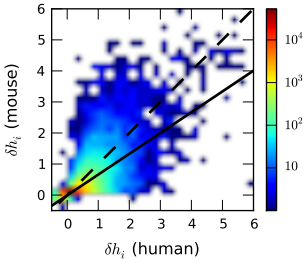

Supplement: Figure S5 — Conservation of orthologous sites. Comparison between inferred values for roughly 80000 orthologous sites using human or mouse as reference species shown as density plot. Solid line indicates regression (Pearson , slope ), dashed line diagonal. (PDF) [file pcbi.1003860.s005.pdf]

**A**

conserved sites

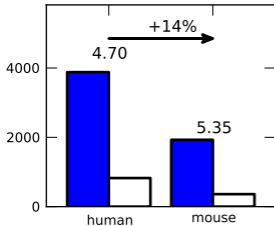**B**

correlated pairs

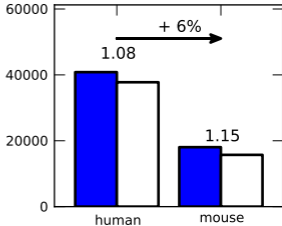

Supplement: Figure S6 — Analysis of statistical power. To assess statistical power of our method, we compare results for a 46-species alignment to human to results using a 60-species alignment to mouse. (A) shows that significantly conserved 7mer and 8mer sites (at a log-likelihood-ratio of , compare Fig. 2C (2)) for miRNA seed matches (blue) and control seeds (white). Signal-to-noise ratio is indicated on top of the bars and increases by 14% when the number of species increases by 30%. (B) Significantly correlated site pairs at a log-likelihood cutoff of as in Fig. 2C (5). Signal-to-noise ratio increases by 6% when increasing the number of species. (PDF) [file pcbi.1003860.s006.pdf]

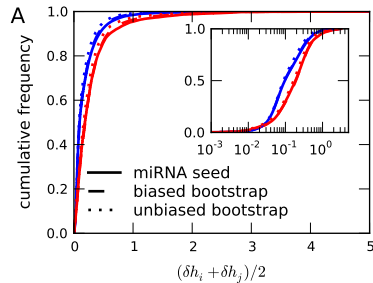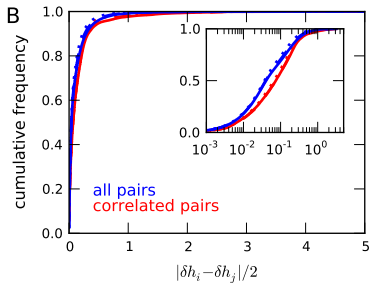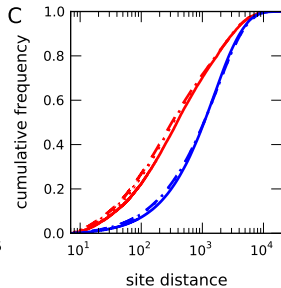

Supplement: Figure S7 — Control seeds. Generating pairs of control seed matches entails selecting control seeds with similar conservation as miRNA seeds (here for the data shown in Fig. 2C ). (A) We compare the histograms for the average conservation of each pair of control seeds (dotted) against pairs of actual miRNA seeds (solid), and use a biased bootstrap to enrich for pairs with similar conservation (dashed, on top of solid). Correlated pairs (red) are on average more conserved than this ensemble. We also checked that the histograms for the difference in conservation (B), and for the site distance (C) are matched. (PDF) [file pcbi.1003860.s007.pdf]

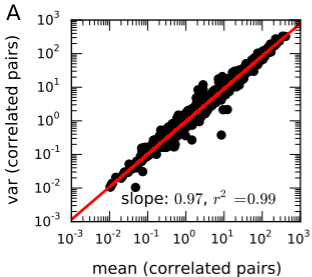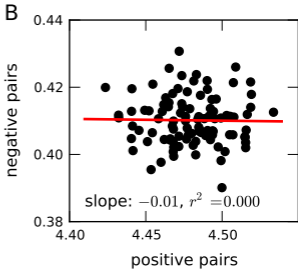

Supplement: Figure S8 — Statistics of correlated site pair occurrence. (A) the number of correlated site pairs for control seeds in the same 3′UTR behaves like a Poisson variable where the mean equals the variance (each dot is a 3′UTR; linear regression on log values). (B) the mean numbers of positively or negatively correlated site pairs per 3′UTR are not correlated (each dot is one bootstrap sample). (PDF) [file pcbi.1003860.s008.pdf]
