# Supplementary material for: Exploring the miRNA Regulatory Network Using Evolutionary Correlations
Source: PLoS Comput Biol. 2014 Oct 9;10(10):e1003860. doi: 10.1371/journal.pcbi.1003860 (PMC4191876; doi:10.1371/journal.pcbi.1003860)
Supplement: Text S1 — Supplementary methods. (PDF) [file pcbi.1003860.s014.pdf]

Exploring the miRNA regulatory network using evolutionary correlations – Supplementary Methods

Benedikt Obermayer and Erel Levine

Kmer conservation statistics

All our inferences are made conditional on a seed match present in the genome of the reference species, and the background model therefore does not need to account for factors influencing the presence of seed matches, such as sequence composition bias and/or different 3'UTR lengths. However, the 3'UTR of each gene has a possibly unique conservation pattern with respect to the set of species where it is alignable and the overall level of sequence conservation, and we take care not to average out this variability. Hence, we define $n_s^{K,g}$ as the number of (overlapping) Kmers in the 3'UTR of gene g that are identically present in the reference species s^* and another species s (such that $n_{s^*}^{K,g} = L_g - K$ where L_g is the length of the 3'UTR in the reference species). Similarly, we define $n_{s,t}^{K,g}$ as the number of Kmers that are present in the reference species s^* and two other species s and t (such that $n_{s^*,s}^{K,g} = n_s^{K,g}$). The conservation frequency of Kmer sites in a single species or a pair of two species is then defined as $f_s^{K,g} = n_s^{K,g}/n_{s^*}^{K,g}$ and $f_{s,t}^{K,g} = n_{s,t}^{K,g}/n_{s^*}^{K,g}$, respectively. To obtain better statistics and reduce noise, we average these values over a set of genes with similar conservation pattern. Specifically, we use a procedure similar to a moving-window average by defining smoothed marginals as

$$\bar{f}_s^{K,g} = \frac{\sum_{g'\in\mathcal{G}(g)} n_s^{K,g'}}{\sum_{g'\in\mathcal{G}(g)} n_{s^*}^{K,g'}} \quad \text{and} \quad \bar{f}_{s,t}^{K,g} = \frac{\sum_{g'\in\mathcal{G}(g)} n_{s,t}^{K,g'}}{\sum_{g'\in\mathcal{G}(g)} n_{s^*}^{K,g'}}, \tag{S1}$$

where $\mathcal{G}(g)$ is the set of 100 genes g' with the most similar conservation profile of their 3'UTRs (i.e., minimal difference $\sum_s (f_s^{K,g} - f_s^{K,g'})^2 / \sum_s (f_s^{K,g})^2$). Note that after averaging we set $\bar{f}_s^{K,g} = 0$ and $\bar{f}_{s,t}^{K,g} = 0$ if the 3'UTR of gene g is not alignable in species s or t, in order to avoid penalizing missing values in observed conservation patterns.

Background model

Our model is now set up in order to reproduce these marginals measured on the leaves of the vertebrate phylogenetic tree. Intuitively, we would describe the gain and loss of target sites along the phylogeny with a phylogenetic Markov model using parameters that would be coarse-grained from established DNA substitution rates [1]. However, such a model is not adequate for this type of data, because (1) the cumulative branch length distances in the vertebrate phylogeny are well beyond the saturation point for neutral substitutions (see the tree in Fig. 1**B**), and (2) the alignment shows a non-negligible and highly non-uniform frequency of gaps (see Fig. 1 in Text S1). These gaps are a consequence not only of local insertions and deletions, but also of large-scale genome rearrangements and an expansion of 3'UTR length in mammals. In addition, gaps also reflect non-biological factors such as the sequencing depth, assembly quality, and alignability of the different genomes. Finally, the gap structure is possibly different for the

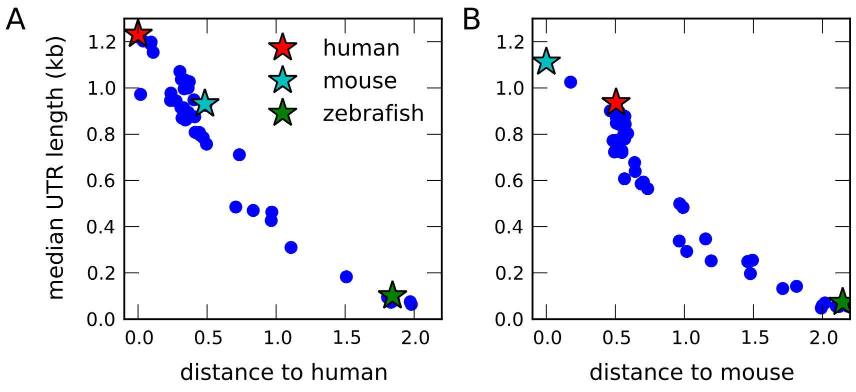

Figure 1: **Statistics of median alignable 3'UTR length. (A)** human as reference species (46way alignment), **(B)** mouse as reference (60way alignment). While the median 3'UTR length of the respective sets of genes (present in human and zebrafish) increases from zebrafish to mammals, most of the effective length differences in the alignments are due to insufficient alignability.

3'UTR of each gene as it might have been completely lost in some species. Hence, the coarse-grained evolution of binding sites cannot be described by an equilibrium Markov model that would require constant gap frequencies along the phylogeny [2] if gaps are treated as additional states. Alternatively, gaps could be treated as missing data, but this would not make use of all available information, for instance when a site is lost (perhaps rendering the surrounding region unalignable) in an otherwise well alignable 3'UTR. Instead, we shift from a standard Markov model to a Markov random field model. As a graphical model, it is formally completely equivalent to a Markov model, and for binary variables it is known as Ising model in statistical physics [3]. Incidentally, the Ising model corresponds to the maximum entropy distribution when single site and pairwise marginals of binary variables are prescribed. Additionally, though, we assume that the interaction topology is given by the vertebrate phylogeny, and marginalize over unobserved ancestral nodes.

For the Markov random field, we introduce phylogenetic parameters $h_s^{K,g}$ and $J_{s,t}^{K,g}$. In an Ising model, these parameters correspond to fields and couplings at nodes s and t, respectively, for observed as well as hidden nodes. The fields $h_s^{K,g}$ are used to prescribe equilibrium frequencies at the leaves of the tree, effectively measure background sequence conservation and alignability in a species-specific way, and are set to zero for the hidden nodes. The couplings (nonzero only for neighboring nodes) take the role of the branch length distances between two nodes and roughly decrease with the logarithm of inverse branch length (measured in units of the neutral substitution rate) [4]. These parameters are simultaneously chosen by solving a nonlinear system of equations such that the measured frequencies and pair frequencies of site occurrence coincide with the expected ones:

$$\bar{f}_s^{K,g} = p_1^{K,g}(\sigma_s^{K,g} = 1) \quad \text{and} \quad \bar{f}_{s,t}^{K,g} = p_2^{K,g}(\sigma_s^{K,g} = 1, \sigma_t^{K,g} = 1). \tag{S2}$$

Formally, these expected marginals are evaluated from the probability distribution for the presence or absence of a Kmer site in the 3'UTR of gene g in species s. We denote these variables by $\sigma_s^{K,g} \in \{1, 0\}$, where the index s includes unobserved ancestral nodes. The joint probability distribution for the entire set $\boldsymbol{\sigma}^{K,g}$ of nodes reads:

$$P_0(\boldsymbol{\sigma}^{K,g}) = \frac{\mathrm{e}^{-H_0^{K,g}(\boldsymbol{\sigma}^{K,g})}}{Z_0^{K,g}}, \tag{S3}$$

where $Z_0^{K,g} = \mathrm{Tr}\, \mathrm{e}^{-H_0^{K,g}}$ is the partition function and the "Hamiltonian" is given by

$$H_0^{K,g}(\boldsymbol{\sigma}^{K,g}) = -\sum_s h_s^{K,g} \sigma_s^{K,g} - \sum_{s<t} J_{s,t}^{K,g} \sigma_s^{K,g} \sigma_t^{K,g}. \tag{S4}$$

Recursion relations to evaluate marginals

Due to the special tree topology induced by the coupling matrix, the marginals used in Eq. (S2) can be efficiently evaluated in linear time using a recursive algorithm known as belief propagation [5]. Defining messages or beliefs $P_{s\to t}(\sigma_s)$, we calculate the marginals as:

$$p_1(\sigma_s) \simeq \mathrm{e}^{-h_s\sigma_s} \prod_{t\in\partial s} \sum_{\sigma_t} \mathrm{e}^{-J_{s,t}\sigma_s\sigma_t} P_{t\to s}(\sigma_t), \tag{S5}$$

where ∂s denotes the neighbors of node s, we omitted the superscripts K and g, and the $\simeq$ symbol means here equality up to normalization, such that $\sum_{\sigma_s} p_1(\sigma_s) = 1$. The messages are computed from the recursion:

$$P_{s\to t}(\sigma_s) \simeq \mathrm{e}^{-h_s\sigma_s} \prod_{u\in\partial s\setminus t} \sum_{\sigma_u} \mathrm{e}^{-J_{s,u}\sigma_u\sigma_s} P_{u\to s}(\sigma_u), \tag{S6}$$

which is evaluated along the tree, in one pass starting from the ancestral nodes outwards to the leaves, and in a second pass inwards starting from the leaves. The pair marginal is computed by conditioning Eq. (S5) on a second leaf being fixed

$$p_2(\sigma_s = 1, \sigma_t = 1) = p_1(\sigma_s = 1|\sigma_t = 1)p_1(\sigma_t = 1), \tag{S7}$$

and Eq. (S5) is used with messages $P_{t\to u}(\sigma_t|\sigma_t = 1) = \delta_{\sigma_t,1}$ at the leaf held fixed set to 1.

Bayesian estimation of preferred conservation

Given a set $\hat{\boldsymbol{\sigma}}_i^{K,g}$ of observations for the presence or absence in different species s (excluding ancestral nodes) of a Kmer site i in the 3'UTR of gene g, the probability of the observed data under the background model reads:

$$P_0(\hat{\boldsymbol{\sigma}}_i^{K,g}) = \frac{\hat{\mathrm{Tr}}\, \mathrm{e}^{-H_0^{K,g}(\boldsymbol{\sigma}_i^{K,g})}}{Z_0^{K,g}}, \tag{S8}$$

where $\hat{\mathrm{Tr}}$ denotes a partial trace over the unobserved ancestral nodes. If now the observed data suggests that the site is conserved more or less widely than expected under the background model, we can quantify this deviation from the null model by including an additional field term δh_i which is constant along the phylogeny:

$$P_i(\hat{\boldsymbol{\sigma}}_i^{K,g}|\delta h_i) = \frac{\hat{\mathrm{Tr}}\, \mathrm{e}^{-H_0^{K,g}(\boldsymbol{\sigma}_i^{K,g}) - \delta H_i(\boldsymbol{\sigma}_i^{K,g})}}{Z_i^{K,g}}, \tag{S9}$$

with the new site-specific partition function $Z_i^{K,g} = \mathrm{Tr}\, \mathrm{e}^{-H_0^{K,g} - \delta H_i}$ and the additional term $\delta H_i(\boldsymbol{\sigma}_i^{K,g}) = -\delta h_i \sum_s \sigma_{i,s}^{K,g}$ in the Hamiltonian. In a Bayesian framework, the optimal value for this additional parameter is then found by maximizing the log likelihood of δh_i given the observed data and the background model:

$$\ln L_i(\delta h_i) = \ln P_i(\hat{\boldsymbol{\sigma}}_i^{K,g}|\delta h_i) - \mu\, \delta h_i^2, \tag{S10}$$

where we include a Gaussian prior to constrain the magnitude of δh_i with parameter μ. The specific numerical choice of $\mu = 0.05$ is largely irrelevant except for an exceedingly small number of sites with nearly perfect conservation where there is not enough variability in the data to obtain a reliable estimate for δh_i; in this way we ensure that $|\delta h_i| \lesssim 5$ stays finite. The log-likelihood ratio $D_i = 2\ln[L_i(\delta h_i) - \ln L_i(0)]$ can be used to quantitatively assess the improvement of the fit to the observed data for site i that inevitably comes with including an additional parameter.

Recursion relations to evaluate Eq. (S10)

The log likelihood Eq. (S10) is evaluated again by an efficient recursion (known as pruning algorithm in statistical genetics [6]). Omitting the superscripts K and g and the site index i, we use Eq. (S9) and write $\ln P(\hat{\boldsymbol{\sigma}}|\delta h) = \ln \hat{\mathrm{Tr}}\, \mathrm{e}^{-H_0-\delta H} - \ln \mathrm{Tr}\, \mathrm{e}^{-H_0-\delta H} = \ln \hat{Z} - \ln Z$ as difference of the logarithm of two partition functions, one obtained in the standard way and the other obtained with leaves fixed to the observed data $\hat{\boldsymbol{\sigma}}$. After computing the messages as in Eq. (S6), the logarithm of the partition function is evaluated as

$$\ln Z = -\sum_{(s,t)} \sum_{\sigma_s,\sigma_t} p_2(\sigma_s,\sigma_t)[(h_s+\delta h)\sigma_s + (h_t+\delta h)\sigma_t + J_{s,t}\sigma_s\sigma_t + \ln p_2(\sigma_s,\sigma_t)] + \sum_s (|\partial s|-1) \sum_{\sigma_s} p_1(\sigma_s)[(h_s+\delta h)\sigma_s + \ln p_1(\sigma_s)], \quad \text{(S11)}$$

where $p_1(\sigma_s)$ is evaluated as in Eq. (S5), $|\partial s|$ is the number of neighbors of node s, and $p_2(\sigma_s,\sigma_t)$, which is only needed for neighboring nodes (s,t), can be directly obtained from the messages as

$$p_2(\sigma_s,\sigma_t) \simeq P_{s\to t}(\sigma_s)\mathrm{e}^{-J_{s,t}\sigma_s\sigma_t} P_{t\to s}(\sigma_t). \quad \text{(S12)}$$

The term $\ln \hat{Z}$ involving the restricted partition function is evaluated in a similar manner, by fixing the messages on the leaves to the observed data: $P_{t\to s}(\sigma_t|\hat{\sigma}_t) = \delta_{\sigma_t,\hat{\sigma}_t}$.

Detection of correlations

We now take this procedure one step further by using two sets of observations $\hat{\boldsymbol{\sigma}}_i^{K_i,g_i}$ and $\hat{\boldsymbol{\sigma}}_j^{K_j,g_j}$ for a pair of sites i and j of possibly different type in the 3'UTRs of two genes g_i and g_j. Under the background model, these conservation patterns should be independent, and this still holds after we quantified their preferential conservation by computing additional fields δh_i and δh_j independently. To account for the possible appearance of significant correlations between the conservation patterns for these sites, we define a more general joint probability distribution for composite variables $S_{ij,s} = (\sigma_{i,s}^{K_i,g_i}, \sigma_{j,s}^{K_j,g_j})$ which take four possible values (known as Potts spins in statistical physics):

$$\mathcal{P}_{ij}(\hat{\boldsymbol{S}}_{ij}|\delta h_i, \delta h_j, \delta h_{ij}) = \frac{\hat{\mathrm{Tr}}\, \mathrm{e}^{-\mathcal{H}_0(\boldsymbol{S}_{ij})-\delta\mathcal{H}(\boldsymbol{S}_{ij})}}{\mathcal{Z}_{ij}}, \quad \text{(S13)}$$

where now the background model for the new variables is given by $\mathcal{H}_0(\boldsymbol{S}_{ij}) = H_0^{K_i,g_i}(\boldsymbol{\sigma}_i^{K_i,g_i}) + H_0^{K_j,g_j}(\boldsymbol{\sigma}_j^{K_j,g_j})$ and the composite correlation term $\delta\mathcal{H}(\boldsymbol{S}_{ij}) = -\delta h_i \sum_s \sigma_{i,s}^{K_i,g_i} - \delta h_j \sum_s \sigma_{j,s}^{K_j,g_j} - \delta h_{ij} \sum_s \sigma_{i,s}^{K_i,g_i}\sigma_{j,s}^{K_j,g_j}$ introduces a coupling between the two observations as a measure for their correlation. The partition function is of course $\mathcal{Z}_{ij} = \mathrm{Tr}\, \mathrm{e}^{-\mathcal{H}_0(\boldsymbol{S}_{ij})-\delta\mathcal{H}(\boldsymbol{S}_{ij})}$. No correlation, i.e., independent conservation, means that $\delta h_{ij} = 0$, and in this case we find that $\mathcal{P}_{ij}(\hat{\boldsymbol{S}}_{ij}|\delta h_i, \delta h_j, 0) = P_i(\hat{\boldsymbol{\sigma}}_i|\delta h_i)P_j(\hat{\boldsymbol{\sigma}}_j|\delta h_j)$ factorizes as expected.

The corresponding log likelihood for the correlation parameter δh_{ij} and single site parameters δh_i and δh_j reads

$$\ln \mathcal{L}_{ij}(\delta h_i, \delta h_j, \delta h_{ij}) = \ln \mathcal{P}_{ij}(\hat{\boldsymbol{S}}_{ij}|\delta h_i, \delta h_j, \delta h_{ij}) - \mu(\delta h_i^2 + \delta h_j^2) - \gamma \delta h_{ij}^2, \tag{S14}$$

which includes another Gaussian prior on the magnitude of δh_{ij} with parameter $\gamma = 0.1$. Again, the exact numerical value of the parameter γ is largely arbitrary except for very few borderline cases where it affects the magnitude of the fitted δh_{ij}, which is not in the focus of our attention. The log-likelihood ratio for the pair inference reads $\mathcal{D}_{ij} = 2\ln[\mathcal{L}_{ij}(\delta h_i, \delta h_j, \delta h_{ij}) - L_i(\delta h_i) - L_j(\delta h_j)]$ and measures by how much the simultaneous fit to two sets of observations improves the independent fits from the first step. Note that the maximization here is performed with respect to three independent parameters, since δh_i and δh_j are re-estimated as well: in the independent estimate, their values include contributions from possible interactions between site i and all other sites. The new estimates for δh_i, however, are not consistent: pair inference between one site i and different sites k would yield different new estimates for the same δh_i. A consistent approach would require to fit all interaction coefficients δh_{ij} simultaneously, which is not feasible with the limited number of genome sequences. Hence, we do not use the new values δh_i and regard δh_{ij} merely as a measure for correlation (not interaction), because indirect contributions to the observed correlations have not been removed.

Recursion relations to evaluate Eq. (S14)

For the evaluation of Eq. (S14), we write again $\ln \mathcal{P}_{ij}(\hat{\boldsymbol{S}}_{ij}|\delta h_i, \delta h_j, \delta h_{ij}) = \ln \hat{\mathcal{Z}}_{ij} - \ln \mathcal{Z}_{ij}$, and use the equivalent of Eq. (S11):

$$\begin{aligned}\ln \hat{\mathcal{Z}}_{ij} = -\sum_{(s,t)} \sum_{S_{ij,s}, S_{ij,t}} \mathcal{P}_2(S_{ij,s}, S_{ij,t})[\mathcal{H}_s^h(S_{ij,s}) + \mathcal{H}_t^h(S_{ij,t}) + \mathcal{H}_{s,t}^J(S_{ij,s}, S_{ij,t}) + \ln \mathcal{P}_2(S_{ij,s}, S_{ij,t})] \\ + \sum_s (|\partial s| - 1) \sum_{S_{ij,s}} \mathcal{P}_1(S_{ij,s})[\mathcal{H}_s^h(S_{ij,s}) + \ln \mathcal{P}_1(S_{ij,s})].\end{aligned} \tag{S15}$$

Here, we split the field and coupling terms in the composite Hamiltonian $\mathcal{H}_0 + \delta\mathcal{H}$ as follows

$$\mathcal{H}_s^h(S_{ij,s}) = -(h_s^{K_i,g_i} + \delta h_i)\sigma_{i,s}^{K_i,g_i} - (h_s^{K_j,g_j} + \delta h_j)\sigma_{j,s}^{K_j,g_j} - \delta h_{ij}\sigma_{i,s}^{K_i,g_i}\sigma_{j,s}^{K_j,g_j}, \tag{S16a}$$

$$\mathcal{H}_{s,t}^J(S_{ij,s}, S_{ij,t}) = -J_{s,t}^{K_i,g_i}\sigma_{i,s}^{K_i,g_i}\sigma_{i,t}^{K_i,g_i} - J_{s,t}^{K_j,g_j}\sigma_{j,s}^{K_j,g_j}\sigma_{j,t}^{K_j,g_j}. \tag{S16b}$$

The new marginals are derived from

$$\mathcal{P}_1(S_s) \simeq \mathrm{e}^{-\mathcal{H}_s^h(S_s)} \prod_{t \in \partial s} \sum_{S_t} \mathrm{e}^{-\mathcal{H}_{s,t}^J(S_s, S_t)} \mathcal{P}_{t \to s}(S_t). \tag{S17}$$

Likewise, the pair marginal reads

$$\mathcal{P}_2(S_s, S_t) \simeq \mathcal{P}_{s \to t}(S_s) \mathrm{e}^{-\mathcal{H}_{s,t}^J(S_s, S_t)} \mathcal{P}_{t \to s}(S_t). \tag{S18}$$

Finally, the messages $\mathcal{P}_{s \to t}(S_s)$ are computed from the recursion

$$\mathcal{P}_{s \to t}(S_s) \simeq \mathrm{e}^{-\mathcal{H}_s^h(S_s)} \prod_{u \in \partial s \setminus t} \sum_{S_u} \mathrm{e}^{-\mathcal{H}_{s,u}^J(S_s, S_u)} \mathcal{P}_{u \to s}(S_u). \tag{S19}$$

Implementation notes

For a two-state variable $\sigma_s \in \{0, 1\}$, the normalization factors omitted in Eqs. (S5) and (S6) do not need to be explicitly calculated. For numerical reasons, it is advantageous to redefine

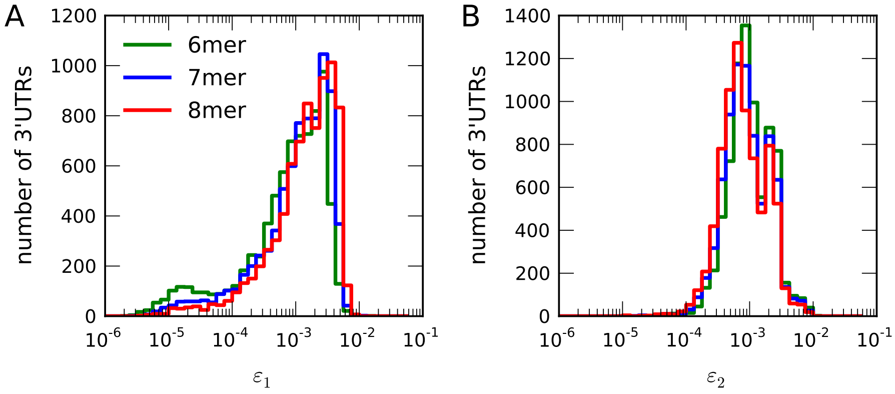

Figure 2: **Performance of the background model.** Histogram of relative errors $\varepsilon_1 = \sqrt{\sum_s (f_s - \pi_s)^2/M}$ of conservation frequencies f_s **(A)** and $\varepsilon_2 = \sqrt{\sum_{s<t}(c_{s,t} - \chi_{s,t})^2/(M(M-1)/2)}$ of pair correlations $c_{s,t} = f_{s,t} - f_s f_t$ **(B)** over 7723 different 3'UTRs and for different site types, respectively. M is the number of species in the alignment ($M = 46$ with human as reference).

$x_s = 2\sigma_s - 1$ which implies $x = \pm 1$. The corresponding fields are given by $\tilde{h}_s = \frac{1}{2}h_s + \frac{1}{4}\sum_t J_{s,t}$, and the couplings are rescaled as $\tilde{J}_{s,t} = \frac{1}{4}J_{s,t}$. Then, Eq. (S5) reads:

$$p_1(x_s = 1) = \left[1 + \mathrm{e}^{-2[\tilde{h}_s + \sum_{t\in\partial s} \mathrm{atanh}(\tanh \tilde{J}_{s,t} \tanh m_{t\to s})]}\right]^{-1} \tag{S20}$$

where simplified messages $m_{s\to t} = \frac{1}{2}\ln\frac{P_{s\to t}(x_s=1)}{P_{s\to t}(x_s=-1)}$ absorbing the normalization factors are derived from the recursion

$$m_{s\to t} = \tilde{h}_s + \sum_{u\in\partial s\setminus t} \mathrm{atanh}(\tanh \tilde{J}_{s,u} \tanh m_{u\to s}). \tag{S21}$$

In order to fit the parameters $h_s^{K,g}$ and $J_{s,t}^{K,g}$ of the background model, we use the Levenberg-Marquardt algorithm for nonlinear least squares problems with a box constraint on fields and couplings to avoid numerical under- and overflow: the fields are required to be smaller than 50 in absolute value, the couplings for neighboring nodes are forced to be positive (larger than e^{-25}), yet smaller than 25. Also, we fit pair correlations $c_{s,t} = f_{s,t} - f_s f_t$ for $s \neq t$ instead of pair marginals and rescale the terms for single and pair marginals to emphasize the importance of reproducing phylogenetic correlations and not just site conservation patterns in different species independently. Altogether, the algorithm solves

$$\min_{h,J} \frac{\sum_s (f_s - \pi_s(h,J))^2}{\sum_t f_t^2} + \frac{\sum_{s<t}(c_{s,t} - \chi_{s,t}(h,J))^2}{\sum_{u<v} c_{u,v}^2}, \tag{S22}$$

where $\pi_s(h,J) = p_1(\sigma_s = 1|h,J)$ and $\chi_{s,t}(h,J) = p_2(\sigma_s = 1, \sigma_t = 1|h,J) - \pi_s(h,J)\pi_t(h,J)$ using Eq. (S20). We recover the measured frequencies f_s and correlations $c_{s,t}$ to a root mean squared accuracy of about 10^{-3} (see Fig. 2 in Text S1).

Simpler background models

For the simplified background models used in Fig. 2**A** and **B**, the statistical weight of a configuration factorizes in to independent contributions from each species, since they are treated independently (using couplings $J_{s,t} \equiv 0$):

$$P_0(\hat{\boldsymbol{\sigma}}_i) = \frac{\mathrm{e}^{-H_0(\hat{\boldsymbol{\sigma}}_i)}}{Z_0} = \prod_s \frac{\mathrm{e}^{h_s\hat{\sigma}_{i,s}}}{1 + \mathrm{e}^{h_s}}. \tag{S23}$$

The parameters h_s are related to the average 7mer conservation frequency for species s in the alignment:

$$h_s = \ln \frac{f_s}{1 - f_s} \text{ where } f_s = \frac{\sum_g n_s^{7,g}}{\sum_g n_{s^*}^{7,g}}. \tag{S24}$$

The model used in Fig. 2**B** simply uses a gene-specific value

$$h_s^g = \ln \frac{f_s^g}{1 - f_s^g} \text{ where } f_s^g = \frac{\sum_{g' \in \mathcal{G}(g)} n_s^{7,g'}}{\sum_{g' \in \mathcal{G}(g)} n_{s^*}^{7,g'}}. \tag{S25}$$

If the alignment for the 3'UTR of gene g is empty, we set $f_s^g = 0$ and use a regularized value $h_s^g = -10$; likewise, since $f_{s^*}^g = 1$ we use $h_{s*}^g = 10$.

The probability of the data under the model with additional field term δh_i is given by:

$$P_i(\hat{\boldsymbol{\sigma}}_i | \delta h_i) = \prod_s \frac{\mathrm{e}^{(h_s + \delta h_i)\hat{\sigma}_{i,s}}}{1 + \mathrm{e}^{h_s + \delta h_i}}. \tag{S26}$$

If all species are treated the same (meaning $f_s \equiv f_0$ and therefore $h_s \equiv h_0$), and if regularization terms are ignored, the maximum likelihood estimate for δh_i can be calculated analytically:

$$\ln P_i(\hat{\boldsymbol{\sigma}}_i | \delta h_i) = (h_0 + \delta h_i) \sum_s \hat{\sigma}_{i,s} - M \ln \left(1 + \mathrm{e}^{h_0 + \delta h_i}\right) \tag{S27}$$

$$\rightarrow \quad \delta h_i = \ln \frac{f_i(1 - f_0)}{f_0(1 - f_i)} \text{ with } f_i = M^{-1} \sum_s \hat{\sigma}_{i,s}. \tag{S28}$$

This means that δh_i is a simple log-odds ratio, where f_i is the observed frequency of site i in the alignment and f_0 the expected one given the background model, with M the number of species. Similarly, defining $f_{ij} = M^{-1} \sum_s \hat{\sigma}_{i,s} \hat{\sigma}_{j,s}$, we get for the maximum likelihood estimate of the correlation score

$$\delta h_{ij} = \ln \frac{f_{ij}(1 - f_i - f_j - f_{ij})}{(f_i - f_{ij})(f_j - f_{ij})}. \tag{S29}$$

Comparison between directed and undirected graphical models

Undirected and directed graphical models are very similar in their mathematical structure. The former are more general, but the latter are often used for more common phylogenetic Markov models. For a directed graph like the one depicted in Fig. 3 in Text S1, the joint probability distribution for the set of binary variables $\boldsymbol{x}$ is given by:

$$p_{\text{directed}}(\boldsymbol{x}) = p_0 \prod_{(s,\pi_s)} p(x_s | x_{\pi_s}), \tag{S30}$$

where p_0 is one single equilibrium frequency and the product runs over edges (s, π_s) between a node s and its parent π_s. For the conditional probability $p(x_s|x_{\pi_s})$, we could choose a simple exponential function of the branchlength $\ell(s, \pi_s)$:

$$p(x_s | x_{\pi_s}) = x_{\pi_s} \mathrm{e}^{-\ell(s,\pi_s)} + p_0 \left[1 - \mathrm{e}^{-\ell(s,\pi_s)}\right]. \tag{S31}$$

In contrast, an undirected model on the same tree is formulated as

$$p_{\text{undirected}}(\boldsymbol{x}) = \frac{1}{Z} \prod_s \varphi(x_s) \prod_{(s,t)} \psi(x_s, x_t), \tag{S32}$$

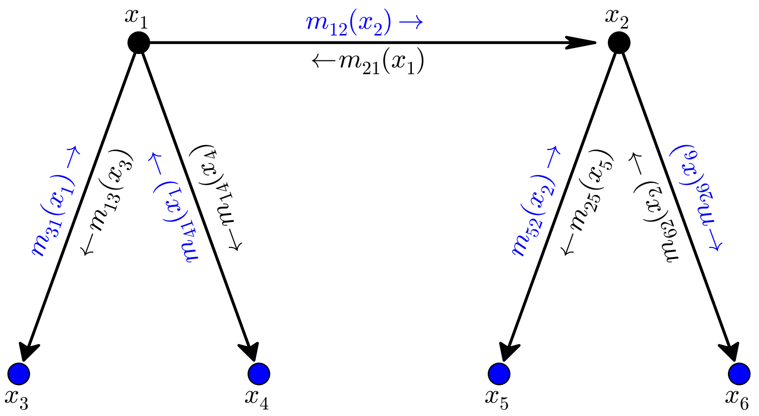

Figure 3: Recursion relations on graphical models. Marginal probability distributions are calculated using messages $m_{st}(x_t)$ summarizing information from upstream nodes (here shown in blue for calculating the marginal distribution $p(x_6)$). Calculation of all marginals requires computing all pairwise messages (black). Adapted from Ref. [7].

where Z is a normalization constant. Our expressions for the node and edge functions $\varphi(x_s)$ and $\psi(x_s, x_t)$, respectively, are inspired by statistical physics but could be chosen differently:

$$\varphi(x_s) = \mathrm{e}^{h_s x_s} \quad \text{and} \quad \psi(x_s, x_t) = \mathrm{e}^{J_{s,t} x_s x_t}. \tag{S33}$$

Notably, both models permit efficient evaluation of a few key quantities such as single-site marginals. For instance, using the tree given in Fig. 3 in Text S1, the marginal probability of x_6 would be calculated in a directed model as follows:

$$p(x_6) = \sum_{x_1}\sum_{x_2}\sum_{x_3}\sum_{x_4}\sum_{x_5} p_0 p(x_6|x_2)p(x_5|x_2)p(x_2|x_1)p(x_3|x_1)p(x_4|x_1). \tag{S34}$$

When (arbitrarily) choosing x_1 as root, this can be rearranged and progressively evaluated as follows:

$$\begin{aligned} p(x_6) &= \sum_{x_2} p(x_6|x_2) \sum_{x_5} p(x_5|x_2) \sum_{x_1} p(x_2|x_1) p_0 \sum_{x_3} p(x_3|x_1) \sum_{x_4} p(x_4|x_1) \\ &= \sum_{x_2} p(x_6|x_2) \sum_{x_5} p(x_5|x_2) \sum_{x_1} p(x_2|x_1) p_0 m_{31}(x_1) m_{41}(x_1) \\ &= \sum_{x_2} p(x_6|x_2) m_{52}(x_2) m_{12}(x_2) \\ &= m_{26}(x_6). \end{aligned} \tag{S35}$$

Here, we defined the messages $m_{st}(x_t)$ that are sent from node s to node t and incorporate all information about the states of upstream nodes (see Fig. 3 in Text S1). These messages would be recursively calculated as

$$m_{st}(x_t) = \sum_{x_s} p(x_t|x_s) \prod_{u\in\partial s\setminus t} m_{us}(x_s), \tag{S36}$$

with $\partial s\setminus t$ denoting all direct neighbors of node s excluding t. This is the well-known sum-product algorithm. To compute all marginal distributions, the entire set of messages shown in Fig. 3 in Text S1 is required.

In an undirected model on the same graph, the site marginal could be evaluated via an essentially identical recursion. Here we would proceed as follows

$$\begin{aligned}
p(x_6) &= \frac{1}{Z}\sum_{x_1}\sum_{x_2}\sum_{x_3}\sum_{x_4}\sum_{x_5}\varphi(x_6)\varphi(x_2)\varphi(x_5)\varphi(x_3)\varphi(x_4)\varphi(x_1) \\
&\qquad\qquad \times \psi(x_1,x_2)\psi(x_6,x_2)\psi(x_3,x_1)\psi(x_4,x_1)\psi(x_5,x_2) \\
&= \frac{1}{Z}\varphi(x_6)\sum_{x_2}\varphi(x_2)\psi(x_6,x_2)\sum_{x_5}\varphi(x_5)\psi(x_5,x_2)\sum_{x_1}\varphi(x_1)\psi(x_1,x_2) \\
&\qquad\qquad \times \sum_{x_3}\varphi(x_3)\psi(x_3,x_1)\sum_{x_4}\varphi(x_4)\psi(x_4,x_1) \\
&= \frac{1}{Z}\varphi(x_6)\sum_{x_2}\varphi(x_2)\psi(x_6,x_2)\sum_{x_5}\varphi(x_5)\psi(x_5,x_2)\sum_{x_1}\varphi(x_1)\psi(x_1,x_2)m_{31}(x_1)m_{41}(x_1) \\
&= \frac{1}{Z}\varphi(x_6)\sum_{x_2}\varphi(x_2)\psi(x_6,x_2)m_{52}(x_2)m_{12}(x_2) \\
&= \frac{1}{Z}\varphi(x_6)m_{26}(x_6),
\end{aligned} \tag{S37}$$

where the messages are calculated from

$$m_{st}(x_t) = \sum_{x_s}\varphi(x_s)\psi(x_s,x_t)\prod_{u\in\partial s\setminus t} m_{us}(x_s), \tag{S38}$$

and the normalization constant Z is obtained by requiring $\sum_{x_6} p(x_6) = 1$.

Following established approaches in statistical physics and information theory [5], our implementation of the recursions Eq. (S6) is actually slightly different: the messages $P_{s\to t}(x_s)$ are normalized probability distributions, and the associated recursions use an interchanged order of sum and product operations:

$$P_{s\to t}(x_s) \simeq \varphi(x_s)\prod_{u\in\partial s\setminus t}\sum_{x_u}\psi(x_s,x_u)P_{u\to s}(x_u). \tag{S39}$$

References

[1] Huelsenbeck, J. and Crandall, K. (1997) Phylogeny estimation and hypothesis testing using maximum likelihood. *Annu Rev Ecol Syst*, **28**, 437–466.

[2] Rivas, E. and Eddy, S. R. (2008) Probabilistic Phylogenetic Inference with Insertions and Deletions. *PLoS Comput Biol*, **4**, e1000172.

[3] Wainwright, M. J. and Jordan, M. I. (2007) Graphical Models, Exponential Families, and Variational Inference. *FNT in Machine Learning*, **1**, 1–305.

[4] Leuthäusser, I. (1986) An Exact Correspondence Between Eigen Evolution Model And A Two-Dimensional Ising System. *J Chem Phys*, **84**, 1884–1885.

[5] Mezard, M. and Montanari, A. (2009) *Information, Physics, and Computation*. Oxford University Press.

[6] Felsenstein, J. (1981) Evolutionary trees from DNA sequences: a maximum likelihood approach. *J Mol Evol*, **17**, 368–376.

[7] Jordan, M. I. and Weiss, Y. (2002) Graphical Models: Probabilistic Inference. in *The Handbook of Brain Theory and Neural Networks, 2nd edition* (Cambridge MA: MIT Press)
